# Lowering levels of reelin in entorhinal cortex layer II-neurons results in lowered levels of intracellular amyloid-β

**DOI:** 10.1093/braincomms/fcad115

**Published:** 2023-04-06

**Authors:** Asgeir Kobro-Flatmoen, Claudia Battistin, Rajeevkumar Raveendran Nair, Christiana Bjorkli, Belma Skender, Cliff Kentros, Gunnar Gouras, Menno P Witter

**Affiliations:** Kavli Institute for Systems Neuroscience MTFS, NTNU Norwegian University of Science and Technology, Olav Kyrres Gate 9, 7489, Trondheim, Norway; KG. Jebsen Centre for Alzheimer’s Disease, NTNU, 7489, Trondheim, Norway; Kavli Institute for Systems Neuroscience MTFS, NTNU Norwegian University of Science and Technology, Olav Kyrres Gate 9, 7489, Trondheim, Norway; Kavli Institute for Systems Neuroscience MTFS, NTNU Norwegian University of Science and Technology, Olav Kyrres Gate 9, 7489, Trondheim, Norway; Kavli Institute for Systems Neuroscience MTFS, NTNU Norwegian University of Science and Technology, Olav Kyrres Gate 9, 7489, Trondheim, Norway; Kavli Institute for Systems Neuroscience MTFS, NTNU Norwegian University of Science and Technology, Olav Kyrres Gate 9, 7489, Trondheim, Norway; Kavli Institute for Systems Neuroscience MTFS, NTNU Norwegian University of Science and Technology, Olav Kyrres Gate 9, 7489, Trondheim, Norway; Mohn Research Center for the Brain, NTNU, 7489, Trondheim, Norway; Institute of Neuroscience, University of Oregon, 97401, Eugene, OR, USA; Experimental Dementia Research Unit, Department of Experimental Medical Science, Lund University, 221 84 Lund, Sweden; Kavli Institute for Systems Neuroscience MTFS, NTNU Norwegian University of Science and Technology, Olav Kyrres Gate 9, 7489, Trondheim, Norway; KG. Jebsen Centre for Alzheimer’s Disease, NTNU, 7489, Trondheim, Norway

**Keywords:** Alzheimer’s disease, molecular interaction, disease onset, animal model

## Abstract

Projection neurons in the anteriolateral part of entorhinal cortex layer II are the predominant cortical site for hyper-phosphorylation of tau and formation of neurofibrillary tangles in prodromal Alzheimer’s disease. A majority of layer II projection neurons in anteriolateral entorhinal cortex are unique among cortical excitatory neurons by expressing the protein reelin. In prodromal Alzheimer’s disease, these reelin-expressing neurons are prone to accumulate intracellular amyloid-β, which is mimicked in a rat model that replicates the spatio-temporal cascade of the disease. Two important findings in relation to this are that reelin-signalling downregulates tau phosphorylation, and that oligomeric amyloid-β interferes with reelin-signalling. Taking advantage of this rat model, we used proximity ligation assay to assess whether reelin and intracellular amyloid-β directly interact during early, pre-plaque stages in anteriolateral entorhinal cortex layer II reelin-expressing neurons. We next made a viral vector delivering micro-RNA against reelin, along with a control vector, and infected reelin-expressing anteriolateral entorhinal cortex layer II-neurons to test whether reelin levels affect levels of intracellular amyloid-β and/or amyloid precursor protein. We analysed 25.548 neurons from 24 animals, which results in three important findings. First, in reelin-expressing anteriolateral entorhinal cortex layer II-neurons, reelin and intracellular amyloid-β engage in a direct protein–protein interaction. Second, injecting micro-RNA against reelin lowers reelin levels in these neurons, amounting to an effect size of 1.3–4.5 (Bayesian estimation of Cohen’s *d* effect size, 95% credible interval). This causes a concomitant reduction of intracellular amyloid-β ranging across three levels of aggregation, including a reduction of Aβ42 monomers/dimers amounting to an effect size of 0.5–3.1, a reduction of Aβ prefibrils amounting to an effect size of 1.1–3.5 and a reduction of protofibrils amounting to an effect size of 0.05–2.1. Analysing these data using Bayesian estimation of mutual information furthermore reveals that levels of amyloid-β are dependent on levels of reelin. Third, the reduction of intracellular amyloid-β occurs without any substantial associated changes in levels of amyloid precursor protein. We conclude that reelin and amyloid-β directly interact at the intracellular level in the uniquely reelin-expressing projection neurons in anteriolateral entorhinal cortex layer II, where levels of amyloid-β are dependent on levels of reelin. Since amyloid-β is known to impair reelin-signalling causing upregulated phosphorylation of tau, our findings are likely relevant to the vulnerability for neurofibrillary tangle-formation of this entorhinal neuronal population.

## Teaser

Kobro-Flatmoen *et al*. report that entorhinal layer II-neurons, which uniquely express reelin, undergo early accumulation of amyloid-β in the context of Alzheimer’s disease. They further show that in these neurons, reelin and amyloid-β42 directly interact, and that lowering reelin levels causes a concomitant reduction of amyloid-β across three levels of aggregation.

## Graphical Abstract

**Graphical Abstract:**
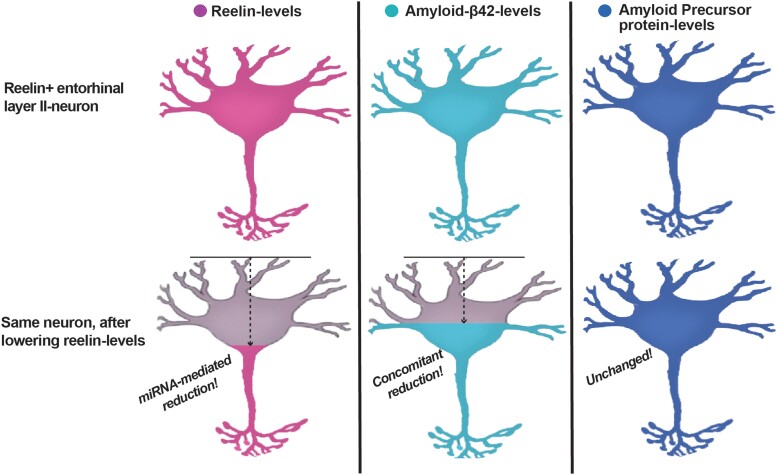

## Introduction

Alzheimer’s disease is a neurodegenerative brain disease that leads to dementia. Two proteins are central in efforts to understand the disease, namely amyloid-β (Aβ) and tau.^1^ It was recently suggested that a key lack in our understanding of the disease, which prevents therapeutic developments, is how these two proteins aberrantly interact to trigger the disease.^2^ To reach a better understanding of these potential interactions, it is necessary to study the initial pathological events that involve specific subsets of neurons.

Recent studies on large human cohorts uncovered evidence that the common, sporadic form of Alzheimer’s disease starts with increased Aβ in the brain, which leads to hyper-phosphorylated tau (*p*-tau) and eventually formation of neurofibrillary tangles (NFTs).^3,4^ This is in line with several prior findings in humans. First, familial, genetically determined forms of the disease are driven by increased levels of total Aβ, or an increase of the more aggregation-prone Aβ42 peptide relative to Aβ40.^5^ Second, Down syndrome leads to severe Alzheimer-pathology by middle age in subjects carrying an added copy of chromosome 21 containing the *amyloid precursor protein (APP)-gene,*^6^ owing to overexpression of APP and a consequent overproduction of Aβ. Third, a genetic variant in humans that results in less Aβ protects against Alzheimer’s disease.^7^

Within the Alzheimer’s disease-research field, the role for Aβ, even during prodromal stages, is most often ascribed to its fibrillar- and deposited extracellular forms, colloquially referred to as Aβ-plaques.^1,8^ In broad strokes, emerging Aβ-plaques notionally damage nearby synapses in a way that induces *p*-tau. Then, as the degree of hyper-phosphorylation reaches beyond a currently unknown threshold, *p*-tau detaches from and disrupts the integrity of microtubules. A consequent degeneration of the affected axon terminals follows, and the now detached *p*-tau translocates or misallocates to somatodendritic compartments, where it builds up and fibrillates into NFTs.^8^ Such a role for Aβ-plaques is however difficult to reconcile with the evidence. Aβ-plaques arise first in the neocortex, typically in the medial orbitofrontal and posterior cingulate areas, quickly followed by the precuneus.^9^ But the cortical onset of NFTs is predominantly restricted to the entorhinal cortex (EC), more specifically, the anteriolateral portion of the entorhinal cortex (alEC), in neurons residing superficially in layer II (LII).^10–14^ The presumed relationship between Aβ-plaques and NFT-onset thus implies that alEC layer II-neurons have projections forming synapses with neurons in the above-mentioned cortical regions. However, findings in rodents^15,16^ and primates^17–22^ show that projections from EC to the medial orbitofrontal cortex, posterior cingulate cortex, and the precuneus, originate predominantly in layer V of EC and only very sparsely or not at all in LII.^16,23–25^

Converging evidence from studies of the human brain using live imaging,^9,26^ immunohistochemistry^27–30^ and biochemistry,^31–36^ supported by experimental results from rodent models^37–40^ and cell models,^37,41^ point to a role for Aβ in non-fibrillated forms in the onset of Alzheimer’s disease. Such a role likely involves interference with cellular homeostatic and metabolic processes^42^ that are neuron-type specific,^43^ implying that the effect of Aβ upon a given population of neurons is not generalizable to all neurons. Using a transgenic mutated human APP-based rat model, we uncovered evidence in support of this notion in that cortical neurons start to accumulate intracellular Aβ (iAβ) up to 8 months before the model develops any Aβ-plaques. In EC, this early accumulation of iAβ restricts to a sub-population of LII-neurons, and these constitute projection neurons that express the glycoprotein reelin. Analysing human subjects with early NFT-pathology (Braak stages I–V) provided corroborating data.^38^ This raised our interest in the role of reelin in the onset of Alzheimer’s disease.

The expression of reelin in projection neurons is atypical for cortex,^44^ but in EC LII, reelin-expressing neurons originate the main projections to the hippocampus.^45^ Reelin is likely crucial to memory formation, and upon binding to its main receptor in brain, ApoER2, triggers a signalling cascade that enhances glutamatergic transmission.^46^ The effect was demonstrated by single injections of reelin into the ventricles of mice, which increased hippocampal dendritic spine density, long-term potentiation (LTP) and memory-dependent task-performance.^47^ Corollary findings showed that reduced levels of reelin associate with impaired memory-dependent task-performance.^48^

A possible interaction between reelin and Aβ was first shown by adding synthetic Aβ42 to cultured SH-SY5Y neuroblastoma cells, causing a dose-dependent increase of the 180-kDa reelin fragment without changing reelin messenger RNA levels.^49^ Further studies on Alzheimer’s disease-subjects revealed that reelin extracted from cortex tends to be of higher molecular mass than the active, signalling-competent form,^50^ and that oligomeric Aβ and reelin co-immunoprecipitate.^51^

Alongside enhancement of glutamatergic transmission as outlined above, the binding of reelin to ApoeR2 triggers a parallel signalling cascade that culminates with potently inhibiting the activity of glycogen synthase kinase 3β (GSK3β).^52–54^ As GSK3β is required to induce long-term depression,^55^ the inhibitory effect of reelin upon GSK3β may help to facilitate LTP. Another crucial role of GSK3β is as one of the main kinases that phosphorylates tau.^56^ Of direct relevance to this are studies showing that an interaction with Aβ impairs the signalling-capacity of reelin, which impairs the inhibitory control exerted by reelin-signalling upon the activity of GSK3β. This leads to constitutive tau phosphorylation by GSK3β, and, importantly, this whole sequence of events is known to result in *p*-tau.^50,52–54,57^

This body of evidence led us to design a comprehensive set of experiments to test whether the selective vulnerability of reelin-expressing alEC LII-neurons to accumulate iAβ is linked to their expression of reelin. In the present study, we show that in reelin-expressing alEC LII-neurons, reelin and iAβ42 engage in a direct protein–protein interaction, and that selectively lowering levels of reelin expression leads to a concomitant reduction of several forms of iAβ. The reduction of iAβ occurs without any substantial associated changes in levels of human APP and occurs prior to any plaque pathology in the rat model.

Our findings have implications for understanding the onset of Alzheimer’s disease. Increased levels of Aβ occur very early in the initiating phase of the disease in reelin-expressing alEC LII-neurons.^38^ The resulting interaction between reelin and Aβ very likely impairs the signalling-capacity of reelin, which, as outlined above, can result in *p*-tau. It is furthermore very likely that, in Alzheimer’s disease, the reelin-expressing alEC LII-neurons, which project to the hippocampal formation, are the first *cortical neurons* to die.^58,59^ Our results allow for an attractive hypothesis that places reelin in the sequence of changes in functional pathways in reelin-expressing alEC LII-neurons resulting in the initiation of Alzheimer’s disease.

## Materials and methods

### Experimental design

We bred and used the McGill-R-Thy1-APP homozygous transgenic rat model, which carries a transgene containing human *APP*751 with the Swedish double and Indiana mutations expressed under the murine Thy1.2 promoter, referred to as AD-rats.^39^ All protocols are approved by the Norwegian Animal Research Authority, complying with the European Convention for the Protection of Vertebrate Animals used for Experimental and Other Scientific Purposes.

Animals were genotyped using quantitative PCR (qPCR).^60^ We used genomic DNA isolated from ear tissue with a High Pure PCR Template Preparation Kit (Roche Diagnostics, Switzerland). The transgene was detected using RT2 qPCR Primer Assays from Qiagen (Netherlands), with a normalization gene (GAPDH or beta-actin), both with FastStart Universal SYBR Green Master (Roche Diagnostics) on an Applied Biosystems StepOnePlus real-time PCR system (Life Technologies Ltd., Thermo Fisher Scientific, USA). From the qPCR, ΔΔCT values were calculated with a known homozygous sample as reference.^61^

### Adeno-associated viral-vector design

The backbone construct of pAAV-CMV-ᵦglobin-intron-MCS-WPRE-hGH PolyA was made using the DNA sequence of Woodchuck hepatitis virus Post-transcriptional Regulatory Element (WPRE), synthesized and cloned after the multi-cloning site in pAAV-MCS (Agilent USA, #240071). We then made the control construct pAAV-CMV-ᵦglobin-intron-EGFP-WPRE-hGH PolyA by cloning the *EGFP* gene sequence between EcoR1 and BamHI restriction sites in the backbone (*the control vector*). Pre-micro-RNA (miRNA) sequences for knocking-down *reelin gene-*expression were designed (miRNA-Re; RNAi designer tool, Thermo Fisher Scientific, USA) along with different pAAV plasmid constructs expressing miRNA-Re. We engineered pre-miRNA constructs with endogenous murine miR-155 flanking sequences, integrated after the *EGFP* sequence using BamHI and HindIII sites in the pAAV-CMV-ᵦglobin-intron-EGFP-WPRE-hGH PolyA backbone (for exact sequences, see Supplementary material). Positive clones were confirmed by restriction digestion analyses and subsequently by DNA sequencing. Two pAAV plasmid constructs, pAAV-CMV-ᵦglobin-intron-EGFP-miR-RE1 and pAAV-CMV-ᵦglobin-intron-EGFP-miR-RE4 targeting different mouse reelin sequences and exhibiting efficient reelin knockdown in a heterologous cell-culture system based *in vitro* knockdown assay were chosen (*the experimental vector*).

Endotoxin free plasmid maxipreps (#12663, Qiagen) were made for adeno-associated viral (AAV) preparations, with vectors packaged in AAV serotype 2/1 capsids (a mosaic of capsid 1 and 2) and purified using Heparin column affinity purification.^62^ The day before transfection, 7 × 106 AAV 293 cells (#CVCL_6871, Agilent, USA) were seeded in DMEM (# 41965062, Thermo Fisher Scientific) containing 10% foetal bovine serum (FBS) (#16000-044, Thermo Fischer Scientific) and penicillin/streptomycin antibiotics (#15140122, Thermo Fisher Scientific) into 150 mm cell-culture plates. Calcium chloride mediated co-transfection was done with 22.5 µg pAAV-containing the transgene, 22.5 µg pHelper (#240071, Agilent, USA), 11.3 µg pRC (#240071, Agilent, USA) and 11.3 µg pXR1 (NGVB, IU, USA) capsid plasmids. The medium was replaced with fresh 10% FBS containing Dulbecco's Modified Eagle Medium (DMEM), 7 h post-transfection. The cells were scrapped out after 72 h, then centrifuged at 200*×g* and the cell pellet was subjected to lysis using 150 mM NaCl-20 mM Tris pH 8.0 buffer containing 10% sodium deoxycholate. The lysate was then treated with benzonase nuclease High Concentration (#71206-3, Millipore) for 45 min at 37°C. Benzonase treated lysate was centrifuged at 3000*×g* for 15 min and the clear supernatant was then subjected to HiTrap® Heparin High Performance (#17-0406-01, GE) affinity column chromatography using a peristaltic pump (McClure C JOVE 2011). The elute from the Heparin column was then concentrated using Amicon Ultra centrifugal filters (#Z648043, Millipore). The titre of viral stock was determined as approximately 1011 infectious particles/ml.

### Stereotaxic injections

We used 24 animals (10 males, 14 females) for stereotaxic injections, of which we injected 5 bilaterally and 19 unilaterally with an *experimental vector* (*miRNA-Re EGFP virus*) versus a *control vector* (*EGFP-only virus*). We injected all animals a few days past 1 month of age (Supplementary Table 1), when their weight was ∼100 g. We anaesthetized animals using 5% isoflurane gas (Abbott Lab., Cat# 05260-05) in an induction chamber and then immediately transferred the animals to a stereotaxic frame with a mask providing a flow of 1.5–2% isoflurane gas for the full length of the surgery.

To target alEC layer II with high precision, we aligned our coordinates to the brains’ sagittal and transverse sinuses, as their attachment to the brain constitute the most stable anchoring points. Specifically, we horizontally levelled the skull in the stereotaxic frame by lowering the tip of the capillary on top of bregma and then adjusting until lambda was at the exact same horizontal position vis a vis the tip of the capillary. We then aligned the capillary tip to the sagittal sinus at the mid-point between bregma and lambda, and from here moved 3.30 mm laterally, and then caudally until crossing the point where the transverse sinus passes, where the capillary tip was again aligned. From this point, the tip was moved 4.60 mm rostrally, and a further 3.60 mm laterally. At this coordinate, a small hole was drilled and the capillary tip was lowered to the surface of the brain, from which point the tip was lowered 4.50 mm to hit alEC layer II. We then waited for 5 min to alleviate any deflections in the brain tissue, before injecting.

Bilateral injections consisted of one injection of the *experimental vector* into either the left or the right alEC, and one injection of the *control vector* into the contralateral alEC. For the unilateral injections, we injected either the experimental or control vector alternately into the left versus right alEC per animal. The volume of the injections ranged from 300 to 900 nl, and was delivered using a micropump (Drummond Nanoject III, Cat# DRUM3-000-207) with a back-filled ultra-thin glass capillary at 30 nl/min. After injecting, we waited 5 min to allow good uptake before slowly retracting the capillary. We then waited for ∼2 months (Supplementary Table 1) before processing the animals.

### Brain extraction, tissue processing and immunohistochemistry

Animals ranged in age from P54 to P78 at the time of euthanasia (Supplementary Table 1). We anaesthetized animals using isoflurane gas in an induction chamber, followed by intra-peritoneal injection of pentobarbital (Norwegian Pharmacy Association, Cat# 306498). Subsequently, we transcardially perfused animals with a Ringer’s Solution (145 mM NaCl, VWR Int. LLC, Cat# 27800.291; 3.35 mM KCl, Millipore, Cat# 1.04936.1000; 2.38 mM NaHCO_3_, Millipore, Cat# 1.06329.1000), oxygenated to pH ∼6.9, followed by circulation of 4% freshly depolymerized paraformaldehyde (Millipore, Cat# 1.04005.1000) in phosphate buffer (PB: purified de-ionized water with di-sodium hydrogen phosphate dihydrate, Millipore, Cat# 1.37036.500, mixed with sodium di-hydrogen phosphate monohydrate, Millipore, Cat# 1.06346.1000, at 125 mM, pH 7.6; note that this applies to all uses of PB) for 2–3 min. Extracted brains were post-fixed (same fixative) overnight and then placed in a freeze protective solution containing 2% Dimethyl sulfoxide (DMSO) and 20% glycerol (PB with DMSO, VWR Int. LLC, Cat# 23486.297, and glycerol, VWR, Cat# 24387.292) at −20°C until sectioning. Brains were coronally sectioned at 40 μm (double injected) or 30 μm (single injected) with a freezing microtome (Microm HM430, Thermo Fisher Scientific). We collected six (for double injected) or eight (for single injected) series of equally spaced sections, randomly assigned one series to each immunohistochemistry (IHC) experiment, and did all incubations on free-floating sections.

Prior to IHC, all tissue was subject to Heat Induced Antigen Retrieval, by immersion in PB at 60°C for 2 h. We blocked tissue with 5% goat serum (2% for 1D1; Abcam, Cat# AB7481) in PB for 1 h. Subsequently, we did double-IHC labelling with primary antibodies (PA) in PB solution containing either 0.2% Triton X-100 (Millipore, Cat# 1.08603.1000) or 0.4% Saponin (VWR, Cat# 27534.187), all with 5% goat serum, as listed (Supplementary Table 2). Note that we chose antibodies against Aβ to provide good coverage of the pre-plaque aggregation steps, including Aβ42 monomers/dimers (IBL),^63^ Aβ prefibrils (A11)^64^ and Aβ protofibrils (OC).^65^ Processed tissue was mounted on Superfrost™ glass slides (Thermo Fisher Scientific) from a solution of 50 mM tris(hydroxymethyl)aminomethane (Millipore, Cat# 1.08382.1000) with hydrochloric acid, at pH 7.6, and then left to dry overnight before being coverslipped using entellan (Merck KGaA, Cat# 1.07960.0500). Due to a technical issue, we did not obtain reliable data for the second series of IHC-experiments (second set of experiments) involving reelin and Aβ42 monomers/dimers (IBL).

### Proximity ligation assay

We based our proximity ligation assay (PLA) protocol on that for Aβ42 and reelin as described above, with the following adaptations as per the manufacturer’s description (Sigma-Aldrich, DUO92101): brain sections were rinsed three times for 10 min in PB, then five times for 10 min in TBS (50 mM Tris, 150 mM NaCl, pH 8.0) and pre-incubated for 1 h in 10% normal goat serum in TBS-TX (solution of 50 mM Tris, 0.87% sodium chloride and 0.5% Triton X-100). Then, sections were incubated with PA (IBL Aβ42 and reelin, see Supplementary Table 2) in 10% normal goat serum in TBS-TX for 48 h. After rinsing, we blocked endogenous peroxidase activity by incubating the tissue in hydrogen peroxide solution for 10 min at room temperature, followed by rinsing in ‘washing solution A’. The tissue was then mounted onto glass slides. Secondary probes attached to oligonucleotides were then added, and, after rinsing, the oligonucleotides of the bound probes were ligated, amplified and visualized by addition of the detection reagent and substrate solution. We then added the nuclear stain solution provided by the kit, followed by dehydrating the sections by immersing them in increasing concentrations of ethanol, ending with a defatting step in xylene for 5 min. For each PLA-experiment, we ran sets of technical negative controls. These included omission of either or both PA, omission of either or both PLA probes, and omission of all the above.

### Imaging and digital processing

We scanned all tissue at 20 × using a Zeiss Axioscanner (Z.1) with Zen software (2.6, Blue Ed.) under identical settings (488 channel for EGFP viral tag; 546 channel for Aβ/APP; 635 channel for reelin), taking care to determine the optimal dynamic range for our labelled sections. We identified alEC LII (specifically, the dorsolateral and the dorsal intermediate EC) using established criteria.^66^ For all sections with infected neurons in alEC we used the Zen Circle Tool to place a circle around each EGFP-positive neuron with a diameter of ∼20 µm located in the superficial part of layer II (sometimes referred to as layer IIa). This approach ensured reliable inclusion of reelin-expressing alEC LII principal neurons, while avoiding calbindin neurons that are situated deeper (LIIb). We then read out the mean pixel intensity for the 546 and 635 channels of each EGFP-positive neuron in Zen. To measure non-infected neurons, we took the contralaterally matching level of alEC (same distance from the rhinal sulcus) on each section, and used the same circle tool on reelin-expressing neurons (i.e. the 635 channel). We obtained background levels for each animal independently by using measurements of the cerebellar white matter; these measurements were subtracted from the value of each neuron of each animal. We then exported the data to Python Pandas DataFrames to carry out the statistical analyses. Note that animal 24 868 immunolabelled against A11 and reelin was excluded due to a technical error. In total, we quantified the levels of reelin and Aβ (three different forms, IBL, A11 and OC, see above) in 5451 experimentally infected neurons, 4869 control infected neurons and 7350 uninfected neurons, along with quantifying the levels of reelin and hAPP in 2046 experimentally infected neurons, 2352 control infected neurons and 3480 uninfected neurons. The person doing the data collection was blind to the identity of the treatment-groups.

### Statistical analysis of differences in protein levels between conditions

The Supplementary material contains the complete methods for our statistical analyses. In brief, we used the fluorescence level (average pixel intensity) of as a readout of the protein level (reelin versus Aβ/APP) per neuron within each animal after subtracting background levels. As the physiological levels of reelin and iAβ are variable,^37,38^ we used normalized data from the neurons on a *per animal level*, by linearly mapping the fluorescence level in the 0.1 interval for all neurons/animal.^67,68^ We used Bayesian estimation (normalized data) of the parameters of a Student-T distribution for each condition and each animal, with the degrees of freedom parameter shared between the two conditions,^69^ and Monte Carlo sampling to estimate the posterior distribution of the effect size between conditions.^70^ For comparison, Bayesian estimation of the effect size was also performed on the normalized data after randomly assigning the labels of the condition. This analysis was conducted independently on reelin fluorescence data, the three different forms of iAβ, and 1D1.

### Regression analysis of reelin versus iAβ fluorescence levels

We performed Bayesian non-parametric regression^71–73^ treating the reelin level as the independent variable and the level of each single iAβ form per animal and condition (miRNA-Re/control/non-infected) as the dependent variable. We then analysed the Mutual Information between the two variables,^74^ using their joint posterior distribution. For comparison, Bayesian estimation of mutual information between reelin and iAβ was also performed on the normalized data for a specific condition and animal after randomly pairing reelin levels to iAβ levels. Bayesian regression was conducted independently on the three different forms of iAβ and 1D1 from each hemisphere (condition).

## Results

**Figure 1:**
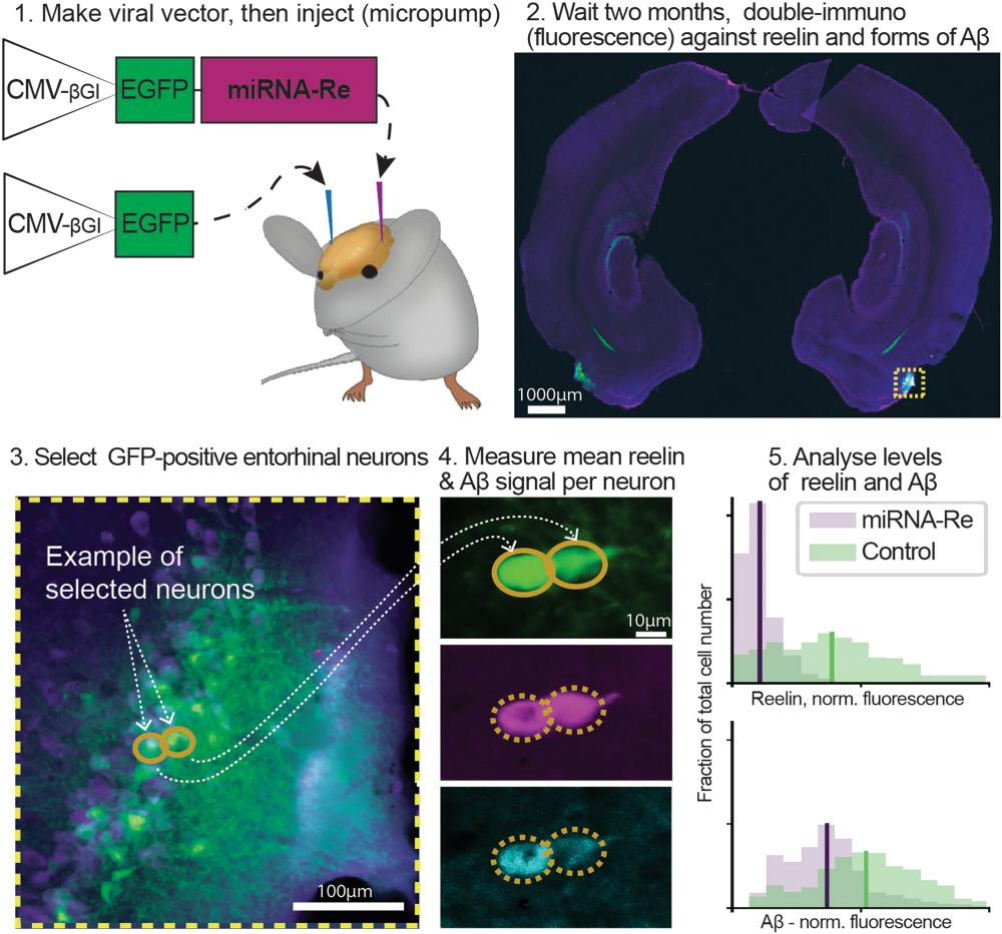
**Overview of experimental set-up to lower reelin in reelin-positive neurons in layer II of the anteriolateral alEC of AD rats.** We used an in-house made AAV that expresses miRNA against reelin (CMB-βGI-EGFP-Reelin-miRNA, in simplified form denoted miRNA-Re) to lower levels of reelin in LII-neurons in alEC (in rodents, also known as dorsolateral entorhinal cortex). The control virus was similar, though lacked the miRNA-Re (CMB-βGI-EGFP, in simplified form denoted control). Tissue sections from all animals were fluorescence-immunolabelled for the presence of reelin and three different forms of iAβ (Aβ42 with IBL, prefibrils with A11 and protofibrils with OC). Levels of fluorescence were quantified using densitometry on automatically scanned sections and the obtained data were normalized and statistically assessed on an animal-by-animal level using Bayesian estimation of the parameters of a Student-T distribution for each condition. Scalebars indicated in images (2–4).

To determine whether the selective vulnerability of reelin-expressing alEC LII-neurons to accumulate iAβ is linked to their expression of reelin, we experimentally assessed the effects on iAβ levels of lowering the expression of reelin in reelin-expressing alEC LII-neurons of Alzheimer’s disease-rats (hereafter referred to simply as model rats), with the use of a viral vector expressing Re-miRNA (Fig. 1).

### Reelin directly interacts with Aβ in reelin-expressing alEC LII-neurons

We first immunolabelled sections of 3-month-old model rats with three different antibodies, selected to capture Aβ across a wide range of configurations prior to formation of mature fibrils that are Aβ plaque-associated. We validated and used a C-terminal specific Aβ42 antibody that detects monomers/dimers (IBLAβ42^63^; Supplementary Fig. 1), and furthermore used a conformation-dependent antibody that detects Aβ prefibrils (A11),^64^ and a second conformation-dependent antibody that detects Aβ protofibrils (OC).^65^ Notably, the binding of prefibrillar forms of Aβ by A11 is mutually exclusive to the binding of protofibrillar forms of Aβ by OC.^65^ In alEC, each of these three antibodies react with intracellular material that is selectively present in reelin-expressing LII-neurons (Supplementary Fig. 2A and B shows example of Aβ42 relative to reelin versus calbindin, respectively), and we observed that each of these forms of Aβ also co-localize with reelin to a high degree at the intracellular level (Supplementary Fig. 2C). This corroborates and extends earlier findings about reelin-expressing alEC LII-neurons where a different set of antibodies against reelin and Aβ were used.^38^

**Figure 2:**
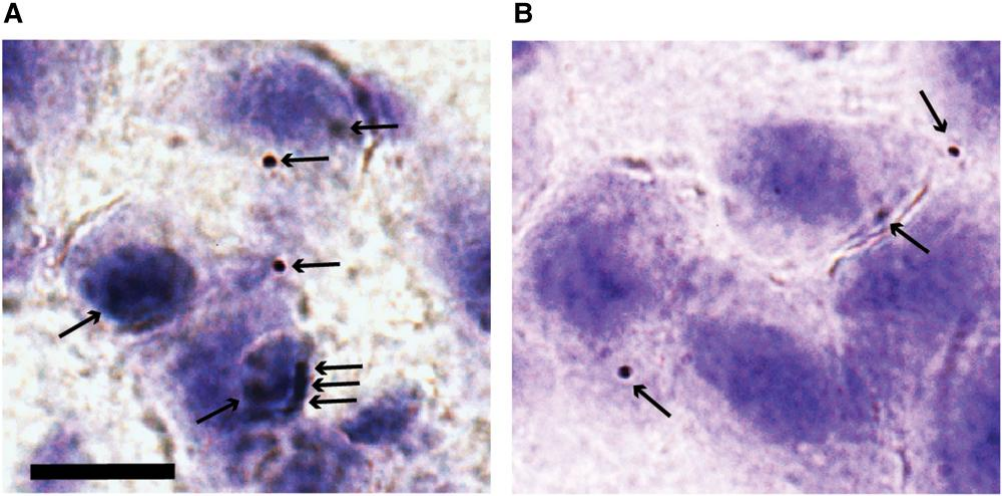
**Proximity ligation assay reveals protein–protein interaction between reelin and intracellular Aβ42 in alEC LII-neurons. (A)** High powered (100×oil) brightfield micrograph of a representative cluster of alEC layer II-neurons from McGill AD rat reveals multiple dark granules (arrows), signalling that intracellular Aβ42 and reelin are within ∼40 nm of each other. **(B)** We find occasional signals also in layer II-neurons from normal (non-AD) littermate controls, confirming that the dramatically increased incidence seen in AD animals reflects their intracellular Aβ42 accumulation. The counterstain is Cresyl-Violet (Nissl-staining). Scalebar in **A**, applicable to **B** as well, equals 10 μm.

To substantiate these confocal results, we used PLA, a highly reliable method to reveal if two proteins are closer than 40 nm.^75^ We found clear and numerous PLA-signals in superficially located alEC LII-neurons in model rats at pre-plaque stages, demonstrating a direct interaction between reelin and Aβ42 in these neurons (Fig. 2A; Supplementary Fig. 3). Because neurons under normal conditions make low amounts of Aβ, including Aβ42,^76^ we decided to test whether a reelin-Aβ42 interaction is detectable also in wild-type rats. Notably, using the same Aβ42 and reelin antibodies as before, on age-matched wild-type Wistar rats, we again find alEC LII-neuron-restricted PLA-signals, albeit at very low levels (Fig. 2B). This latter finding indicates that the tendency of selective accumulation of iAβ42 in reelin-expressing alEC LII-neurons is not an artefact of the transgene-expression of the model rats but likely represents a cell-biological feature of reelin-expressing alEC LII-neurons.

### Lowering reelin in reelin-expressing alEC LII-neurons concomitantly reduces iAβ

We next set out to test whether an interaction exists between the two proteins. To selectively lower reelin in reelin-expressing alEC LII-neurons, we constructed an *experimental vector* carrying miRNA against reelin (CMB-βGI-EGFP-Reelin-miRNA) plus a *control vector* (CMB-βGI-EGFP). We successfully placed selective bilateral injections, the experimental vector on one side and the control vector on the contralateral side, into LII of alEC in five model rats (Fig. 1; Supplementary Fig. 4). To target the earliest possible Alzheimer-related stage of the model rats, after EC has fully developed, we injected animals at ages a few days past 1 month.^77^

**Figure 3:**
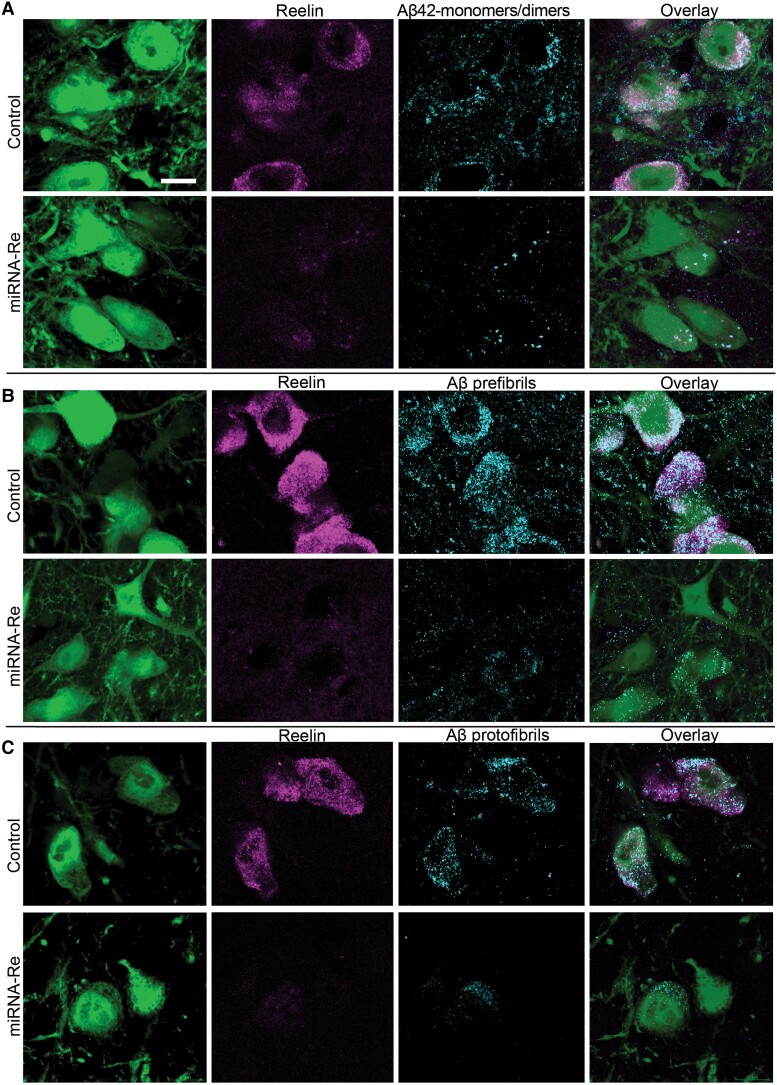
**Lowering levels of reelin in reelin-expressing alEC LII-neurons results in a concomitant reduction of levels of iAβ across three aggregation states.** (**A–C**). Each panel shows confocal optical sections of 0.7 μm thickness to illustrate the effect of the experimental vector (miRNA-Re) relative to control for reelin and (**A**) Aβ42 monomers/dimers, (**B**) Aβ prefibrils and (**C**) Aβ protofibrils. The micrographs were acquired using identical settings. Scalebar in (**A**) is for all micrographs and equals 10 μm.

Confocal images of randomly selected sets of immunolabelled reelin-expressing alEC LII-neurons expressing the experimental vector were compared with images of reelin-expressing alEC LII-neurons at the corresponding dorsoventral position of the contralateral alEC expressing the control vector. The experimental vector efficiently lowered reelin levels relative to the control vector. Furthermore, immunolabelling against iAβ revealed a concomitant reduction for each of the three aggregation states tested, i.e. Aβ42 monomers/dimers, Aβ prefibrils and Aβ protofibrils (Fig. 3A–C).

**Figure 4:**
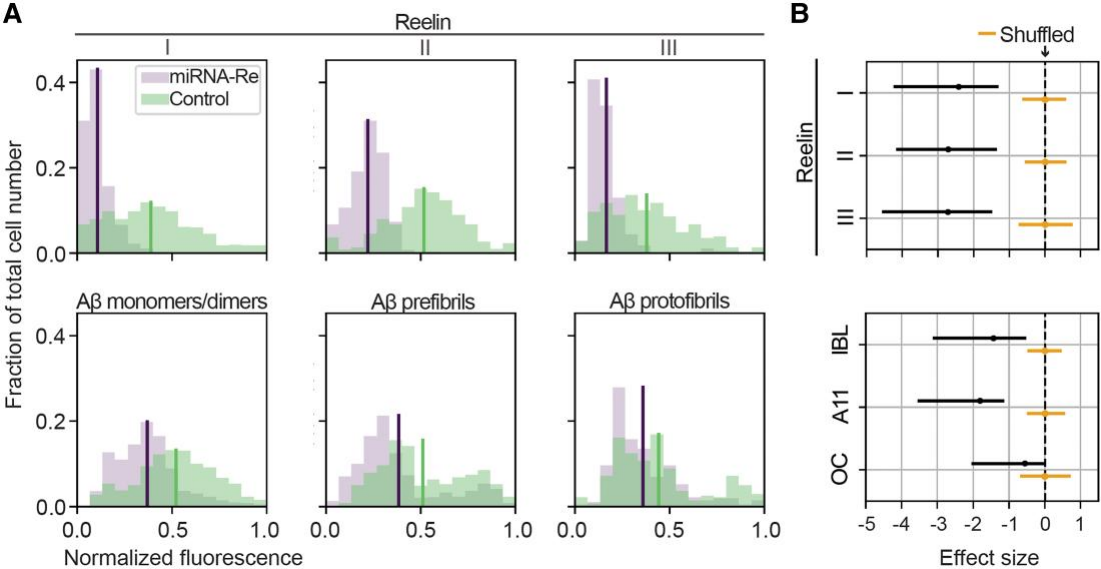
**Quantification of the lowering of reelin and the concomitant reduction of iAβ across three aggregation states in reelin-expressing alEC LII-neurons.** (**A**) Upper row: plots showing levels of reelin as measured by average immunofluorescence per neuron (binned) following injections of the experimental vector (miRNA-Re) to lower reelin levels, relative to a control vector. Lower row: plots showing the resulting effects from lowering reelin levels on levels of three different forms of Aβ, including Aβ monomers/dimers (IBL), Aβ prefibrils (A11) and Aβ protofibrils (OC). Lines indicate the mean of the sample distribution. (**B**) The associated right-side diagrams show the effect of the manipulation for each plot, expressed as posterior Cohen’s *d* effect sizes. Reelin is lowered with a mean effect size of 2.6 [95% credible interval = (1.3–4.5) upper three rows], leading to a concomitant lowering of iAβ that for Aβ42 monomers/dimers (IBL) amounted to a mean effect size of 1.4 [95% credible interval = (0.5,3.1)], while for prefibrils (A11) the mean effect size was 1.8 [95% credible interval = (1.1–3.5)] and for protofibrils (OC) the mean effect size was 0.6 [95% credible interval = (0.05, 2.1)]. Posterior distribution of the mean of the effect size from the shuffled data is indicated around the rightmost dashed line. Solid lines span the 95% credible interval, while the dots represent the mean. *n* for reelin and IBL: miRNA-Re vector 820 neurons, control vector 585 neurons. *n* for reelin and A11: miRNA-Re vector 685 neurons, control vector 579 neurons. *n* for reelin and OC: miRNA-Re vector 365 neurons, control vector 283 neurons. Data are from five double-injected animals (see the ‘Materials and Methods’ section and Supplementary Table 1).

To quantify the efficiency of the lowering of reelin and the concomitant reduction of iAβ in reelin-expressing alEC LII-neurons, we used fluorescence densitometry. The average fluorescence levels for reelin and each of the three forms of iAβ (i.e. three different IHC-procedures) for individual neurons infected with the experimental vector or the control vector were quantified. Bayesian estimation showed that the level of reelin is significantly reduced by a mean effect size of 2.6, ranging from 1.3 to 4.5 (95% credible interval, Cohen’s *d* effect size) for neurons infected by the experimental vector relative to neurons infected by the control vector. This led to a concomitant reduction of iAβ that for Aβ42 monomers/dimers (IBL) amounted to a mean effect size of 1.4 [95% credible interval = (0.5,3.1); experimental vector *n* = 820; control vector *n* = 585], for prefibrils (A11) the mean effect size was 1.8 [95% credible interval = (1.1–3.5); experimental vector *n* = 685, control vector *n* = 579], and for protofibrils (OC) the mean effect size was 0.6 [95% credible interval = (0.05, 2.1); experimental vector *n* = 365, control vector *n* = 283; Fig. 4].

We then inferred a predictive distribution of the levels of Aβ given the reelin level via Bayesian non-parametric regression, using the average fluorescence level measured for each reelin-expressing alEC LII-neuron for each of the three IHC-procedures. This showed that, regardless of whether reelin was lowered or not, the levels of reelin are predictive of the levels of each form of Aβ (monomers/dimers, prefibrils and protofibrils). Bayesian estimation of mutual information indeed reveals a clear dependency between reelin and each form of Aβ, with minimal overlap between the 95% credible interval for the measurements and the shuffle distribution (Supplementary Fig. 5).

We previously reported that levels of Aβ vary in a non-systematic way between the left and right EC of the rat model we use.^60^ We therefore also tested the effects of the experimental versus control vector against neurons in non-injected hemispheres, by placing unilateral injections of either the experimental (9 animals) or the control vector (10 animals) in LII of alEC. Confocal images again showed that the experimental vector efficiently lowered reelin levels relative to the control vector. The amount was quantified by fluorescence levels for reelin and the effect of changing the reelin levels on the fluorescence levels for Aβ prefibrils (A11) and protofibrils (OC) in reelin-expressing alEC LII-neurons. We compared fluorescence levels in neurons infected with the experimental vector, with uninfected reelin-expressing neurons from the corresponding dorsoventral position of the contralateral alEC. As the uninfected neurons do not express green fluorescent protein, we selected these by their expression of reelin. We used the same approach for animals injected with the control vector. For this second set of experiments, the experimental vector reduced reelin by a mean effect size of 2.2 standard deviations [95% credible interval = (1.7, 3.9)]. This led to a concomitant reduction of levels of Aβ prefibrils (A11) with a mean effect size of 1.5 standard deviations [95% credible interval = (0.5, 2.0); experimental vector *n* = 1601, non-infected *n* = 2059], and for levels of protofibrils (OC) the reduction had a mean effect size of 1.3 standard deviations [95% credible interval = (0.6, 1.7); experimental vector *n* = 1980, non-infected *n* = 1892], thus substantiating the findings from the first set of experiments. The control vector led to a mean reduction of reelin amounting to 0.38 standard deviations relative to uninfected neurons. This had no impact on the levels of Aβ (prefibrils, control vector *n* = 2244, non-infected *n* = 2219; protofibrils, control vector *n* = 1178, non-infected *n* = 1180; Supplementary Fig. 6).

The inferred predictive distribution of the Aβ levels given the reelin levels substantiated our findings from the first set of experiments, thus showing that the levels of reelin are predictive of the levels of Aβ prefibrils (A11) and Aβ protofibrils (OC). Bayesian estimation of mutual information again revealed a clear dependency between reelin and both forms of Aβ, with minimal overlap between the 95% credible interval for the measurements and the shuffle distribution (Supplementary Fig. 7).

### The reduction of iAβ by lowering of reelin in reelin-expressing alEC LII-neurons is independent of APP levels

**Figure 5:**
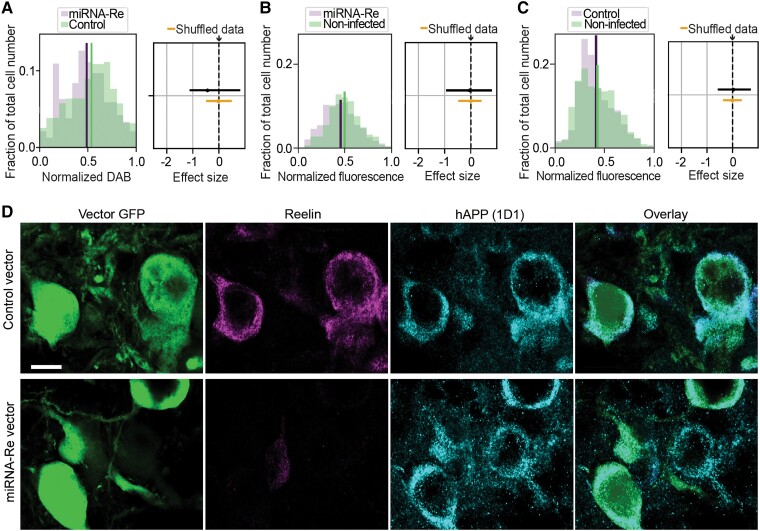
**Reduced accumulation of iAβ, induced by lowering reelin in reelin-expressing alEC LII-neurons, occurs without substantial associated changes in human APP levels.** (**A**) Double-injected animals: left-side plot shows levels of human amyloid precursor protein (hAPP, antibody 1D1), measured using DAB, in neurons infected with the experimental vector (miRNA-Re) to lower reelin levels, compared with contralateral neurons infected with a control vector. Right-side plot shows that the experimental vector does not have any effect on levels of hAPP [mean posterior Cohen’s *d* effect size expressed as standard deviations (SD) = −0.35, 95% credible interval = (−1.1,0.8)]. (**B**) Single-injected animals: left-side plot shows levels of hAPP (1D1), measured using fluorescence, in neurons infected with the experimental vector (miRNA-Re) to lower reelin levels (10 animals), compared with non-infected neurons. Right-side plot indicates that the experimental vector caused a minor reduction of levels of hAPP [mean effect size = 0.05 SD, 95% credible interval = (−0.8,0.9)]. (**C**) Same as for (**B**) but for neurons infected with a control vector (nine animals) and compared with non-infected neurons. Right-side plot shows that the control vector does not have any effect on levels of hAPP [mean effect size = 0.03 SD, 95% credible interval = (−0.5,0.7)]. (**D**) Double-immunofluorescence of reelin-expressing alEC LII-neurons in single-injected animals. Top row: confocal optical sections of 0.7 μm thickness illustrate that upon infection with the control vector, reelin is readily apparent along with hAPP (1D1). Bottom row: infection with the experimental vector (miRNA-Re), which effectively reduces reelin, does not lead to a visible change in levels of hAPP. The micrographs were acquired using identical settings. Scalebar in top left micrograph represents all and equals 10 μm. *n* for 1D1 with DAB: miRNA-Re vector 237 neurons, control vector 156 neurons. *n* for reelin and 1D1 (double-fluorescence): miRNA-Re vector 1809 neurons versus non-infected 1798 neurons, and control vector 2196 neurons versus non-infected 1682 neurons. Data in (**A**) are from five double-injected animals, data in (**B**) are from 19 single side-injected animals, 9 with experimental vector, 10 with control vector (see the ‘Materials and Methods’ section or Supplementary Table 1).

The transgene of the rat model we used drives expression of mutated human APP, and these mutations shift the processing of the human APP to cause pathological amounts of Aβ.^78^ Therefore, any manipulation that changed Aβ levels could stem from changes in human APP levels. To assess whether the effect of lowering reelin upon iAβ resulted from changes in human APP levels, we used a well-validated human APP antibody (1D1)^79^ on tissue from both the first and the second set of experiments. To check whether measurements might be influenced by the use of fluorophores or chromogenic labelling, we used immunoenzyme labelling with 3,3′-diaminobenzidine (DAB) for the material from the first set of experiments, and fluorophores (as used for the above IHC) for the second set of experiments. The results from the first set of experiments using DAB showed that neurons infected with the experimental vector retained the same levels of human APP as neurons infected with the control vector. Data from the second set of experiments substantiate this, as neurons infected with the experimental vector showed only a minor reduction of human APP levels relative to neurons infected with the control vector [mean Cohen’s *d* effect size = 0.1, 95% credible interval = (−0.8, 0.6); numbers of neurons for first set of experiments: experimental vector *n* = 237, control vector *n* = 156; numbers of neurons for second set of experiments: experimental vector *n* = 1809, non-infected *n* = 1798; control vector *n* = 2196, non-infected *n* = 1682; Fig. 5]. Bayesian non-parametric regression shows that, contrary to the case for reelin levels upon Aβ levels, reelin levels are not predictive for levels of human APP. Results from Bayesian estimation of mutual information furthermore indicates a lack of dependency between reelin and human APP (Supplementary Fig. 8).

## Discussion

It is likely that progress in therapeutic approaches against Alzheimer’s disease is prevented by a lack in our understanding of how Aβ and *p*-tau interact to trigger the disease.^2^ In a previous paper, we showed that the major subset of principal neurons in alEC LII, unique in their expression of reelin, are prone to accumulate iAβ.^38^ Our current data show that long before Aβ-plaques start to form, reelin and iAβ42 can structurally associate in these neurons. We further show that selectively lowering the amount of reelin in the reelin-expressing alEC LII-neurons reduces their propensity to accumulate iAβ. This reduction encompasses three levels of Aβ-aggregation, monomers/dimers, prefibrils and protofibrils, and occurs without any substantial associated changes in human APP levels.

Aβ-plaques no doubt play a major role in the context of full-blown Alzheimer’s disease, but the situation is different in the prodromal phase of the disease. Studies on living human subjects show that cerebrospinal fluid levels of Aβ, as measured with flow cytometry using microsphere-based Luminex xMAPTM technology, change before formation of Aβ-plaques, where the latter were revealed by positron emission tomography.^9,26^ Biochemical studies support this since levels of non-fibrillated Aβ predict cognitive decline and neurodegeneration better than the amount of Aβ-plaques,^31–36^ and these studies are corroborated by experimental results obtained both in animal models^37–39^ and neuron cultures.^37,41^ Human post-mortem studies using sensitive immunohistochemical methods are also in line with this, showing that Aβ starts to accumulate intracellularly prior to formation of Aβ-plaques,^27–30^ and that such iAβ-accumulation is striking in EC LII-neurons.^30,38,80–82^ This body of evidence provides a strong argument that non-fibrillated forms of Aβ are highly relevant in the onset of Alzheimer’s disease. But how might reelin play into this?

The reelin–Aβ interaction we report here corroborates an earlier report that the two molecules may directly interact in the brain as well as in cultured cells.^51^ These interactions probably depend on molecular features of the two molecules. Reelin is a self-associating and cysteine-rich protein that contains multiple β-strands.^83,84^ Alongside the potential for β-strands to aggregate, it is worth noting that Aβ-peptides exhibit pathological binding specifically to cysteine-rich domains.^85^ Another relevant factor is that the reelin C-terminus has a strong positive charge,^86^ and Aβ42-peptides have a negative charge.^87^ Recent findings show that neuron-specific increases of proteins that exceed their solubility levels will drive these proteins towards aggregation.^43^ Such an exceeding of solubility levels is likely at play also regarding findings that Aβ causes a dose-dependent increase of non-signalling-competent reelin. The latter observations were made in cell-cultures and substantiated by findings in the human Aβ-affected neocortex,^49,50^ where reelin and oligomeric Aβ were shown to co-immunoprecipitate.^51^ Intriguingly, all of these factors are likely joined in the context of reelin-expressing alEC LII-neurons and so help explain their selective accumulation of iAβ.

The interaction between reelin and Aβ prevents reelin from triggering processing and internalization of ApoER2.^50,51,88^ This ApoER2 processing normally creates an *N*- as well as a C-terminal ApoER2-fragment, both detectable in cerebrospinal fluid. In line with the Aβ-induced impairment of reelin-signalling found in cultured cells, the amounts of these ApoER2-fragments drop by about 50% in cerebrospinal fluid of Alzheimer’s disease-subjects, even though levels of *ApoER2 messenger RNA* as well as full-length ApoER2 remain unaltered.^51,89^ This provides convincing corroborative evidence that an interaction between reelin and Aβ occurs in the brain of Alzheimer’s disease-subjects, and that this impairs the reelin-signalling cascade. Because this signalling cascade works to enhance glutamatergic transmission in the adult brain and promotes LTP,^46,47^ loss of reelin in adulthood predicts deficits in memory functions in mice and monkeys. Such deficits were indeed shown by recent experiments on reelin conditional knockout mice,^48^ and this is further corroborated by findings that an age-related decline in reelin levels in reelin-expressing alEC LII-neurons in monkeys associates with memory impairments.^90^

Besides its role in memory, reelin-signalling through ApoER2 initiates a second signalling cascade mediated by tau-phosphorylating kinases. When initiated, this cascade potently inhibits the activity of one of the main tau-phosphorylating kinases, namely GSK3β.^52–54^ Metabolically active reelin thus regulates the activity of GSK3β such that low levels of reelin result in increased levels of *p*-tau, as demonstrated in congenitally reelin-deficient mice (reeler-mice) that show a strong increase of *p*-tau.^54^ A converse situation is apparent when mating transgenic mice overexpressing reelin, with transgenic mice expressing mutated human tau that normally develop ample *p*-tau by 6 months. The resulting offspring exhibit a dramatic reduction of *p*-tau.^68^ Furthermore, applying reelin exposed to Aβ onto mouse primary neurons results in a 2-fold increase in levels of *p*-tau in the cell-extracts relative to that seen following the addition of pure reelin.^50^ These converging results convincingly show that increased reelin-signalling serves to downregulate the phosphorylation-state of tau. Vice versa, one might thus predict that lowered levels of active reelin, resulting from the molecular interactions between reelin and iAβ42, will result in increased phosphorylation of tau. This is of interest in view of the strong evidence that the predominant cortical onset of NFTs occurs in alEC LII.^10–14^ Moreover, in the rat model, levels of iAβ and reelin in EC LII show a clear gradient, with both being high in neurons situated anteriolaterally, but gradually lower in neurons situated successively more medially.^38^ Future work should address whether these graded levels of iAβ and reelin directly relate to the gradient established for NFTs, which, following their onset in alEC LII, progressively invade more medially situated LII-neurons.^12,14,91^ Moreover, it would also be highly relevant to determine whether or how *p*-tau levels in alEC LII-neurons change in response to artificially lowering levels of reelin.

**Figure 6:**
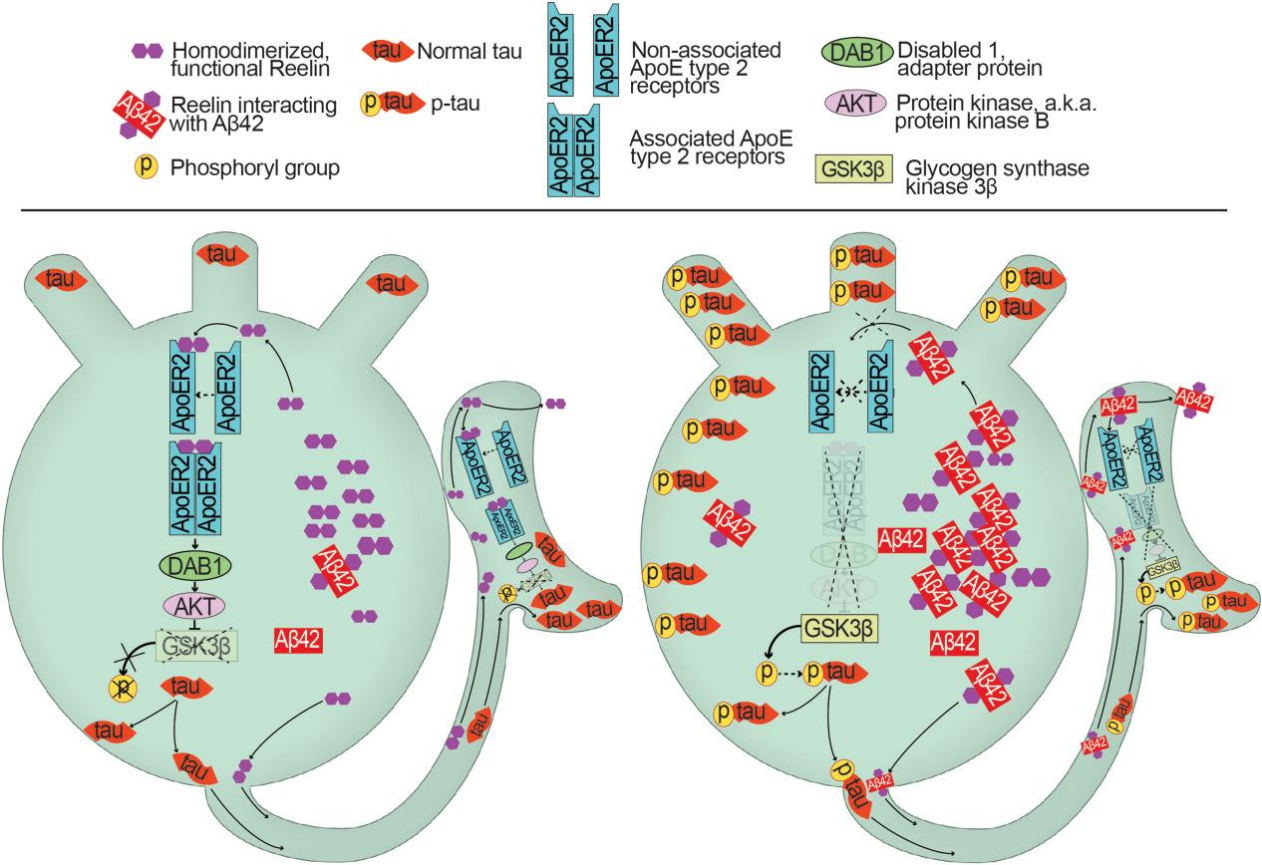
**Schematic representation of a mechanistic model for how reelin-iAβ42 interactions lead to a selective vulnerability in formation of *p*-tau in reelin-expressing alEC LII-neurons.** Left-side cartoon: in a healthy reelin-expressing alEC II-neuron, there is little accumulation of iAβ42. Intact reelin-signalling leads to a low activity level of GSK3β and helps ensure the appropriate phosphorylation-state of tau. This is part of the requirement for tau to remain localized to the proper compartments, with the highest levels being in the axon. Right-side cartoon: as the autophagic machinery becomes less efficient owing to ageing, iAβ42 will tend to accumulate at a disproportional rate in reelin-expressing alEC LII-neurons, due to the high metabolic rate of these neurons. When this happens, iAβ begins pathologically interacting with reelin, further increasing the workload upon, and failure of, the autophagic machinery. The interaction of reelin with iAβ will cause reelin-iAβ42 complexes to form and increasingly lead to signalling-deficient reelin supplanting signalling-competent reelin. The reelin-iAβ42 complexes are unable to bind the ApoER2-receptor to initiate the downstream signalling cascade. This removes the inhibitory signalling upon GSK3β, causing the latter to drive hyper-phosphorylation of tau (*p*-tau), which is misallocated away from the axon and into somatodendritic compartments where NFTs form.

The above findings, alongside our present findings of a pre-plaque interaction between reelin and Aβ in reelin-expressing alEC LII-neurons, allow for an attractive hypothesis about how Aβ and *p*-tau interact to trigger Alzheimer’s disease (Fig. 6). In particular, alEC LII-neurons are thought to have a very high metabolic rate, and, as the autophagic machinery becomes less efficient owing to aging, iAβ will tend to accumulate at a disproportional rate in these neurons.^42^ When this happens, iAβ begins to pathologically interact with reelin, causing further accumulation of iAβ, and further increasing the workload upon, and failure of, the autophagic machinery. The interaction of reelin with iAβ will then increasingly lead to signalling-deficient reelin supplanting signalling-competent reelin. This causes a failure of reelin to bind ApoER2 and a consequent downregulation of the signalling cascades normally controlled by reelin, leading to impairments in entorhinal-hippocampal LTP, and, crucially, a failure to downregulate the activity of GSK3β. The subsequent increased activity of GSK3β leads to increased formation of *p*-tau that finally results in NFTs. The finding that expressing the ɛ4 variant of apolipoprotein, which constitutes the highest risk factor for Alzheimer’s disease aside from ageing, impairs recycling of ApoER2 due to vesicular trapping in the endocytic transport machinery^92^ ties in well with the current hypothesis. This is because one can logically predict that the impaired signalling via the ApoeR2, due to iAβ-induced loss of reelin-signalling, will have the greatest impact in subjects in which the ApoER2 is already compromised by expressing the ɛ4 apolipoprotein variant. The current hypothesis provides a testable prediction: any compound preventing reelin from associating with iAβ in reelin-expressing alEC LII-neurons, which does not in itself trigger production of more Aβ, will prevent or at least substantially reduce the formation of *p*-tau and NFTs.

## Supplementary Material

fcad115_Supplementary_Data

## Data Availability

Upon publication of this manuscript, all data will be made publicly available through the Norwegian University of Science and Technology Open Research Data repository (https://dataverse.no/dataverse/ntnu).

## Abbreviations

Aβ =: amyloid-β
alEC =: anteriolateral entorhinal cortex
APP =: amyloid precursor protein
ApoER2 =: apolipoprotein E receptor 2
DAB =: 3,3'-diaminobenzidine
EC =: entorhinal cortex
GSK3β =: glycogen synthase kinase-3β
iAβ =: intracellular amyloid-β
LII =: layer II
LTP =: long-term potentiation
NFT =: neurofibrillary tangles
*p*-tau =: hyper-phosphorylated tau
