# Supplementary material for: Lowering levels of reelin in entorhinal cortex layer II-neurons results in lowered levels of intracellular amyloid-β
Source: Brain Commun. 2023 Apr 6;5(2):fcad115. doi: 10.1093/braincomms/fcad115 (PMC10120433; doi:10.1093/braincomms/fcad115)
Supplement: fcad115_Supplementary_Data [file fcad115_supplementary_data.docx]

**Supplementary Information for**

Lowering levels of reelin in entorhinal cortex layer II-neurons results in lowered levels of intracellular amyloid-β.

Authors: Asgeir Kobro-Flatmoen, Claudia Battistin, Rajeevkumar Raveendran Nair, Christiana Bjorkli, Belma Skender, Cliff Kentros, Gunnar Gouras, and Menno P. Witter.

Correspondence to: Menno P. Witter (menno.witter@ntnu.no), Olav Kyrres gate 9, 7030 Trondheim, Norway.

Correspondence may also be sent to: Asgeir Kobro-Flatmoen (asgeir.kobro-flatmoen@ntnu.no), Olav Kyrres gate 9, 7030 Trondheim, Norway.

**This PDF file includes:**

Supplementary materials:

Sequence of reelin used for knockdown, and statistical/regression analyses, pp. 2-5 incl. supplementary references

Supplementary Figures 1-8, pp. 6-13.

Supplementary Tables 1-2, pp. 14-15.

**Sequence of reelin used for knockdown.**

We designed multiple candidate microRNAs that target the R3-6 region of murine reelin (Reln), variant 1,NM_011261.2. The pre-microRNA constructs were designed on endogenous murine miR-155 flanking sequences and were cloned after the EGFP sequence using BamHI and HindIII sites in pAAV-CMV-ᵦglobin-intron-EGFP-WPRE-hGH PolyA backbone. The positive clones were confirmed by restriction digestion analyses and subsequently by DNA sequencing. Two pAAV plasmid constructs targeting 5´-TTATTCCAGTTGTCAACCCAA-3´ (miR-RE1) and 5´-TTGATATCGACTGCCTCTCTA-3´(miR-RE4) sequences in mouse Reelin gene, that exhibited efficient Reelin knockdown in a heterologous cell-culture system based *in vitro* knockdown assay were chosen for *in vivo* studies.

Sequences of the two miRs selected for the present study:

miR-RE1

***GGATCC***TGGAGGCTTGCTGAAGGCTGTATGCTGTTGGGTTGACAACTGGAATAAGTTTTGGCCACTGACTGACTTATTCCATGTCAACCCAACAGGACACAAGGCCTGTTACTAGCACTCACATGGAACAAATGGCC***AAGCTT***

Sequences in bold italics are ***BaMHI and HindIII*** restriction sites are used to clone the pre-miR into the vector backbone. Underlined sequence represents the pre-miR, with yellow highlighted region representing antisense target sequence, and blue highlighted region is the sense target sequence. The rest of the sequence in pre-miR is the scaffold derived miR-155.

miR-RE4

***GGATCC***TGGAGGCTTGCTGAAGGCTGTATGCTGTAGAGAGGCAGTCGATATCAAGTTTTGGCCACTGACTGACTTGATATCCTGCCTCTCTACAGGACACAAGGCCTGTTACTAGCACTCACATGGAACAAATGGCC***AAGCTT***

The following two sequences of miRs that showed less efficient knock-down in an vitro assay were not selected for the study:

miRNA2

***GGATCC***TGGAGGCTTGCTGAAGGCTGTATGCTGTTCACCAGAAACCACGACATGGTTTTGGCCACTGACTGACCATGTCGTTTTCTGGTGAACAGGACACAAGGCCTGTTACTAGCACTCACATGGAACAAATGGCC***AAGCTT***

miRNA3

***GGATCC***TGGAGGCTTGCTGAAGGCTGTATGCTGTTCCCAAGGCCATTAAAGTAGGTTTTGGCCACTGACTGACCTACTTTAGGCCTTGGGAACAGGACACAAGGCCTGTTACTAGCACTCACATGGAACAAATGGCC***AAGCTT***

**Statistical analysis of differences in protein levels between conditions.**

We considered the fluorescence level (average pixel intensity) of the protein (reelin vs forms of iAβ), obtained as described (main paper, Imaging and digital processing)*,* of all selected neurons within one animal from both hemispheres (conditions: experimental vector-infected and control vector-infected; experimental vector-infected and non-infected; control vector-infected and non-infected). We then subtracted the background level of the protein for each animal. A few negative values occurred, and these were set to zero. Given the substantial biological variability in the physiological levels of reelin and iAβ^1,2^, data from each single animal and both conditions were normalized, by linearly mapping the fluorescence level in the 0,1 interval^3,4^. We then used the normalized data from each single animal to perform Bayesian estimation of the parameters of a Student-T distribution for each condition, with the degrees of freedom parameter shared between the two conditions^5^. Monte Carlo samples from the posterior distribution of the means ($\mu_{1},\mu_{2}$) and standard deviations${(\sigma}_{1},\sigma_{2}$) for the conditions 1 and 2 were used to estimate the posterior distribution of the effect size^6^, defined as:

$\delta\left( 1,2 \right)=\frac{\mu_{1}-\mu_{2}}{\frac{1}{2}\sqrt{\sigma_{1}^{2}+\sigma_{2}^{2}}}$*.*

Samples of the posterior effect sizes from all single animal models where finally pooled together. We adopted $(-0.4,0.4)$ as region of practical equivalence for the effect size^7^; $\delta\in(-0.6,-0.4) or (0.4,0.6)$ was regarded as a small effect size, while $\delta<-0.6 or >0.6$ as big one. For comparison, Bayesian estimation of the effect size was also performed on the normalized data after randomly assigning the labels of the condition. This analysis was conducted independently on reelin fluorescence data, the three different forms of iAβ, and 1D1.

**Regression analysis of reelin vs iAβ fluorescence levels.**

For all selected cells fluorescence levels (average pixel intensity), obtained as described (main paper, Imaging and digital processing), were normalized at the single protein and animal level, as explained in the preceding section*.* For each animal and condition (experimental vector-infected, control vector-infected, non-infected), we performed Bayesian non-parametric regression^8,9^ treating the reelin level as the independent variable “x” and the level of each single iAβ form as the dependent variable “y”. In our Bayesian hierarchical model the likelihood $p(y|x)$is normal, with a stationary standard deviation ε, while the mean of the Gaussian is modelled as a function f(x) of the independent variable. For the function f(x) we employed a Gaussian process prior with exponential quadratic kernel, which assumes smoothness but allows for a wide range of functional relationships between x and y^10^.

Finally, we sampled from the joint posterior of *f(x)* and ε and we computed the mutual information

$$MI\left[ x;y \right]=\sum_{x,y} p(x)p(y|x){log}_{2}\frac{p(y|x)}{\sum_{x',y} p(x')p(y|x')}$$

between the dependent variable and the independent one^11^. Only for the sake of computing mutual information, “x” was discretized over the range covered by the normalized data for that specific condition, and the probability of “x”, $p(x),$assumed to be flat. Samples of mutual information from single animal models were finally pooled together.

For comparison, Bayesian estimation of mutual information between reelin and iAβ was also performed on the normalized data for a specific condition after randomly pairing reelin levels to iAβ levels. Bayesian regression was conducted independently on the three different forms of iAβ and 1D1 from each hemisphere (condition).

**Supplementary references**

1. Roos TT, Garcia MG, Martinsson I, et al. Neuronal spreading and plaque induction of intracellular Abeta and its disruption of Abeta homeostasis. *Acta Neuropathol*. Oct 2021;142(4):669-687. doi:10.1007/s00401-021-02345-9

2. Kobro-Flatmoen A, Nagelhus A, Witter MP. Reelin-immunoreactive neurons in entorhinal cortex layer II selectively express intracellular amyloid in early Alzheimer's disease. *Neurobiol Dis*. Sep 2016;93:172-83. doi:10.1016/j.nbd.2016.05.012

3. Nakamura T, Oh CK, Liao L, et al. Noncanonical transnitrosylation network contributes to synapse loss in Alzheimer's disease. *Science*. Jan 15 2021;371(6526):eaaw0843. doi:10.1126/science.aaw0843

4. Rossi D, Gruart A, Contreras-Murillo G, et al. Reelin reverts biochemical, physiological and cognitive alterations in mouse models of Tauopathy. *Prog Neurobiol*. Mar 2020;186:101743. doi:10.1016/j.pneurobio.2019.101743

5. Kruschke JK. Bayesian estimation supersedes the t test. *J Exp Psychol Gen*. May 2013;142(2):573-603. doi:10.1037/a0029146

6. Cohen J. *Statistical power analysis for the behavioral sciences*. Academic press; 2013.

7. Kelter R. Analysis of Bayesian posterior significance and effect size indices for the two-sample t-test to support reproducible medical research. *BMC Med Res Methodol*. 2020;20(1):1-18.

8. Martin O. *Bayesian analysis with Python: introduction to statistical modeling and probabilistic programming using PyMC3 and ArviZ*. Packt Publishing Ltd; 2018.

9. Rasmussen C, Williams C. Gaussian Processes for Machine Learning (; Cambridge, MA. MIT Press; 2006.

10. Bernardo J, Berger J, Dawid A, Smith A. Regression and classification using Gaussian process priors. *Bayesian statistics*. 1998;6:475.

11. Cover TM. *Elements of information theory*. John Wiley & Sons; 1999.

**Supplementary figures and tables**

**
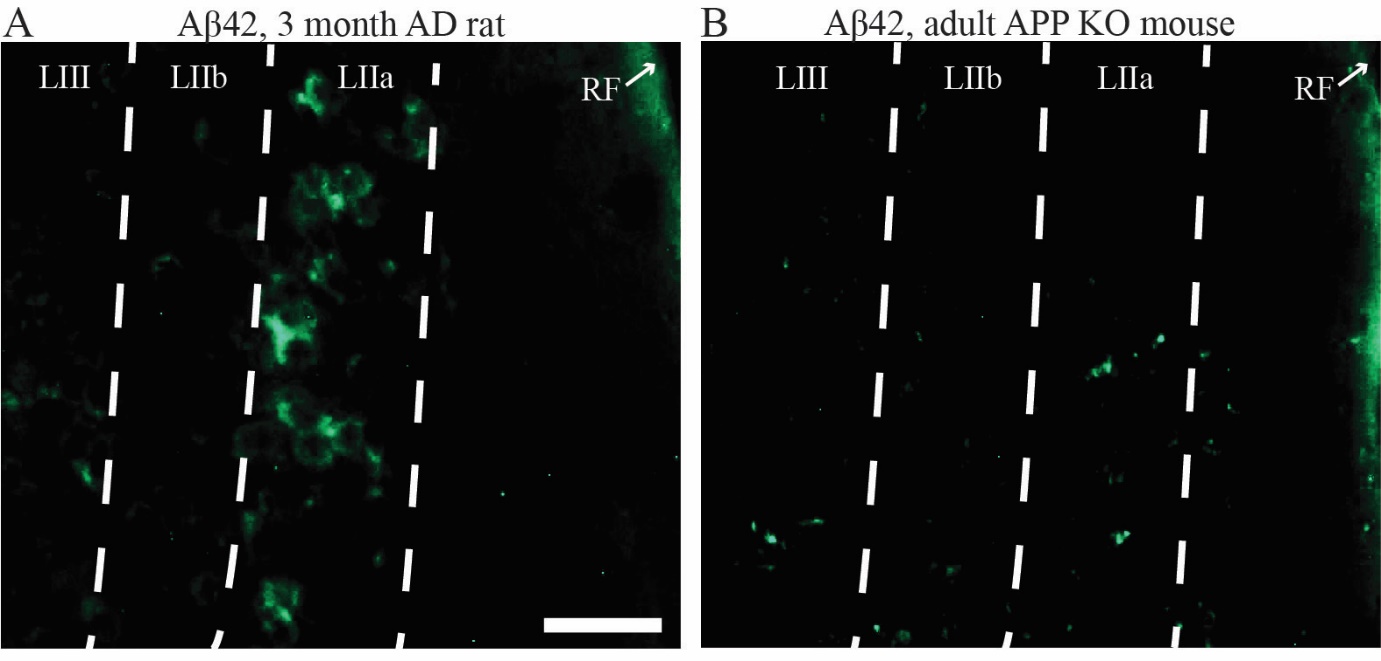
**

**Supplementary figure 1.** Validation of IBL anti Aβ42 antibody. **(A)** Immunolabeling of alEC using the IBL anti Aβ42 antibody on 3-month-old homozygous McGill rats, which is the age at which our analyses were made, reveals clear and consistent labeling of outer LII-neurons (LIIa, i.e. the reelin layer, see Supplementary figure 2). **(B)** Using the same antibody under identical conditions on LEC of adult APP knockout mice, which do not make Aβ, results in an absence of signal, thus substantiating the previously reported specificity of this antibody. Scalebar = 50μm and applies to both (A) and (B).


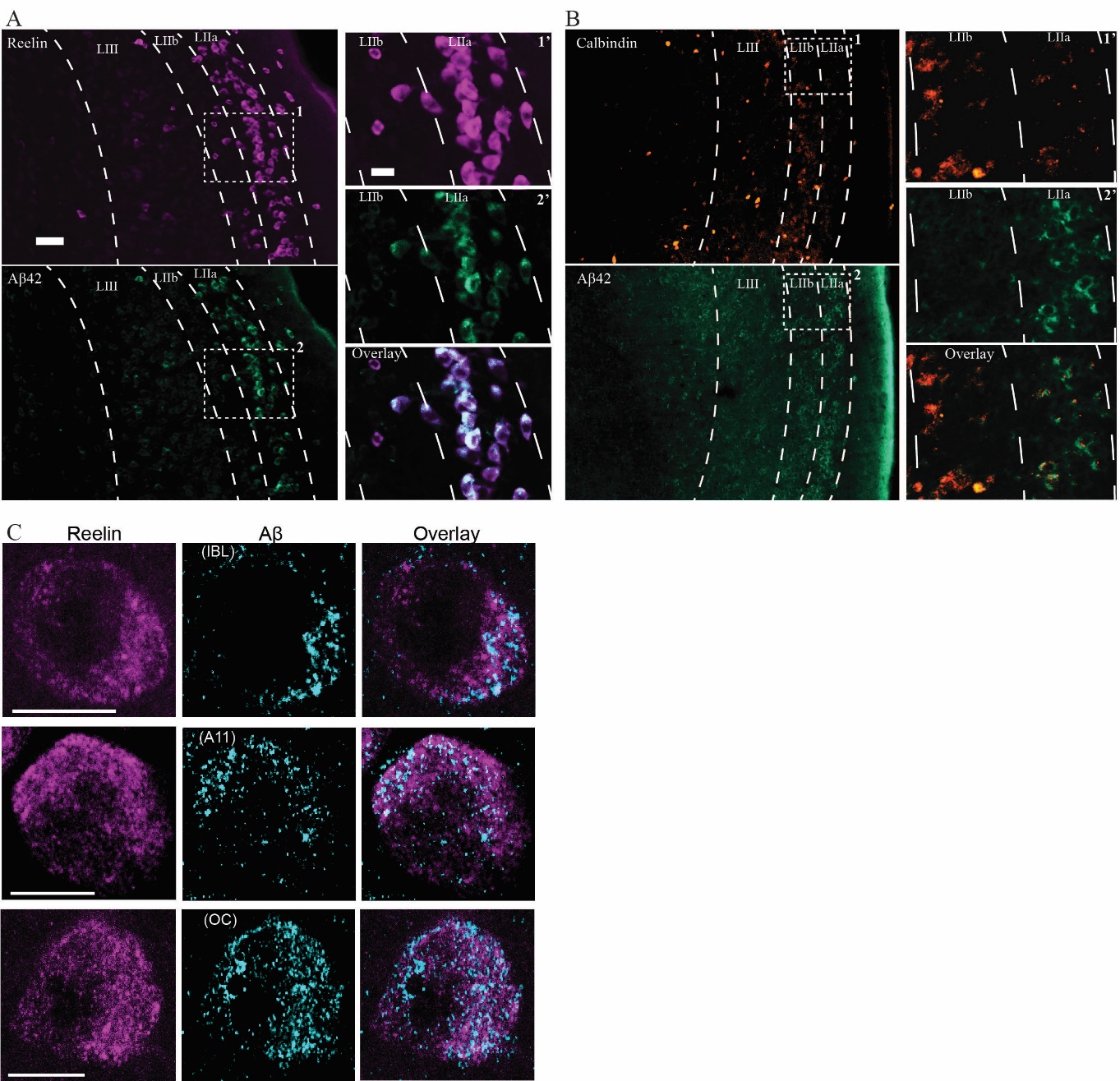


**Supplementary figure 2.** **(A)** Double-immunofluorescence labeling against reelin and iAβ42 monomers/dimers (IBL) in young adult AD rat shows how, in alEC, iAβ-pathology begins in/is restricted to reelin-positive layer II-neurons. **(B)** Double-immunofluorescence labeling against calbindin and iAβ42 monomers/dimers (IBL) in the same rat shows how iAβ-pathology is not present in calbindin-positive neurons. Comparing the right side insets in (A) and (B) reveals the restriction of neurons positive for reelin (A) vs calbindin (B) into two subparts of alEC layer II (reelin in LIIa, calbindin in LIIb). **(C)** Subcellular co-localization between reelin and different forms of iAβ in cytoplasmic granules of reelin-positive alEC LII-neurons in young adult AD rats. Confocal 0.7μm optical sections of double immunohistochemical stains with reelin (left-side) vs three forms of Aβ (middle), including iAβ42 monomers/dimers (IBL; top), iAβ prefibrils (A11; middle), and iAβ protofibrils (OC; bottom). The right side column shows the overlays. Scalebars in (A) = 50μm (same for insets), these also applies to (B). Scalebars in (C) = 10μm.


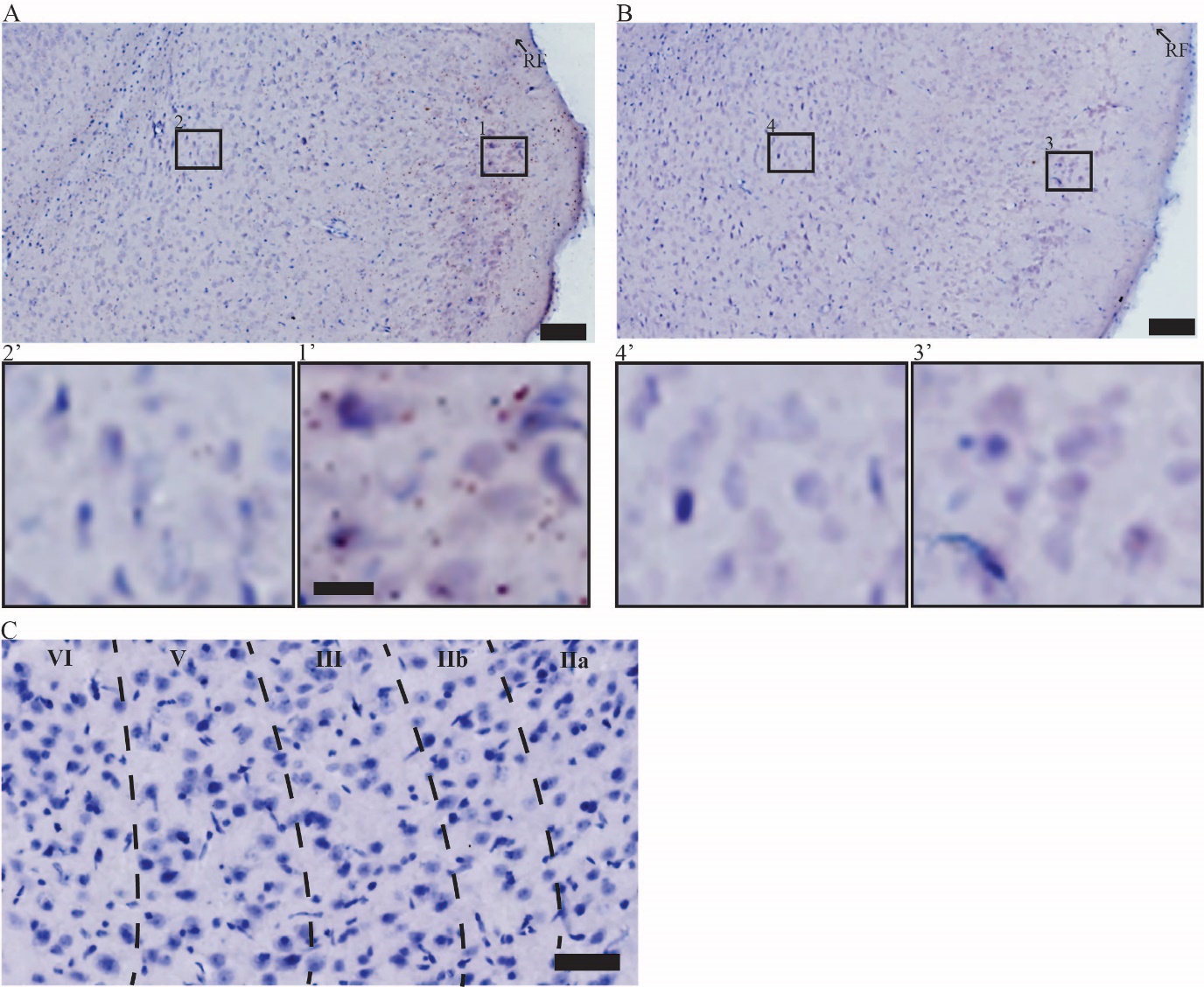


**Supplementary figure 3.** Distribution of Proximity Ligation Assay (PLA)-based interaction between Aβ42 and reelin in the lateral entorhinal cortex (LEC). **(A)** Representative example of PLA-based interaction between Aβ42 and reelin in AD rat, counterstained with Nissl to show the cytoarchitecture. Note the many dark red spots, predominantly in the cytosol, that are particularly numerous in superficial layer II-neurons (inset 1), and then become far less numerous as one moved into layer III, before disappearing when moving into the deep layers (inset 2). **(B)** Control experiment on adjacent section from the same animal, her run without antibodies and PLA-probes, shows complete absence of signal. **(C)** A complete absence of signal also results from running the experiment with the PLA-probes, but in the absence of antibodies. LEC layers are indicated from II-VI. All images were obtained using an axioscanner at 20X. Scalebars in A & B = 100μm (insets, 20μm shown in 1’ represents all insets), C = 50μm.


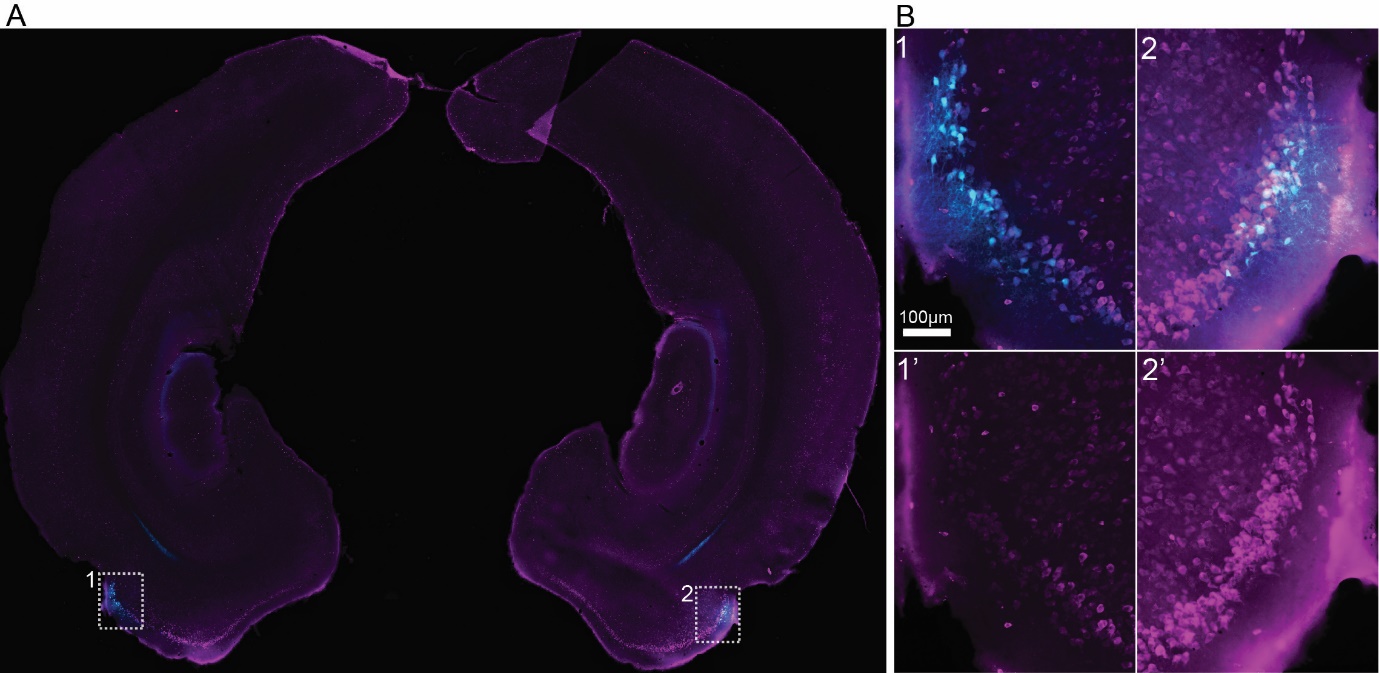


**Supplementary figure 4.** Example of double-injected brain. **(A)** The right anterolateral entorhinal cortex was injected with the control vector, while the left anterolateral entorhinal cortex was injected with the experimental vector (miRNA-Re). Both injection sites and the associated transfection of neurons are labeled due to EGFP-expression (pseudo-colored cyan). **(B)** Higher power image of the insets indicated in A, showing the restriction of the injection (cyan) to the outer part of EC LII (LIIa), and the effect of the experimental vector on the expression of reelin (magenta fluorescence). All imaging settings are identical. Scalebar in (B) = 100 µm.


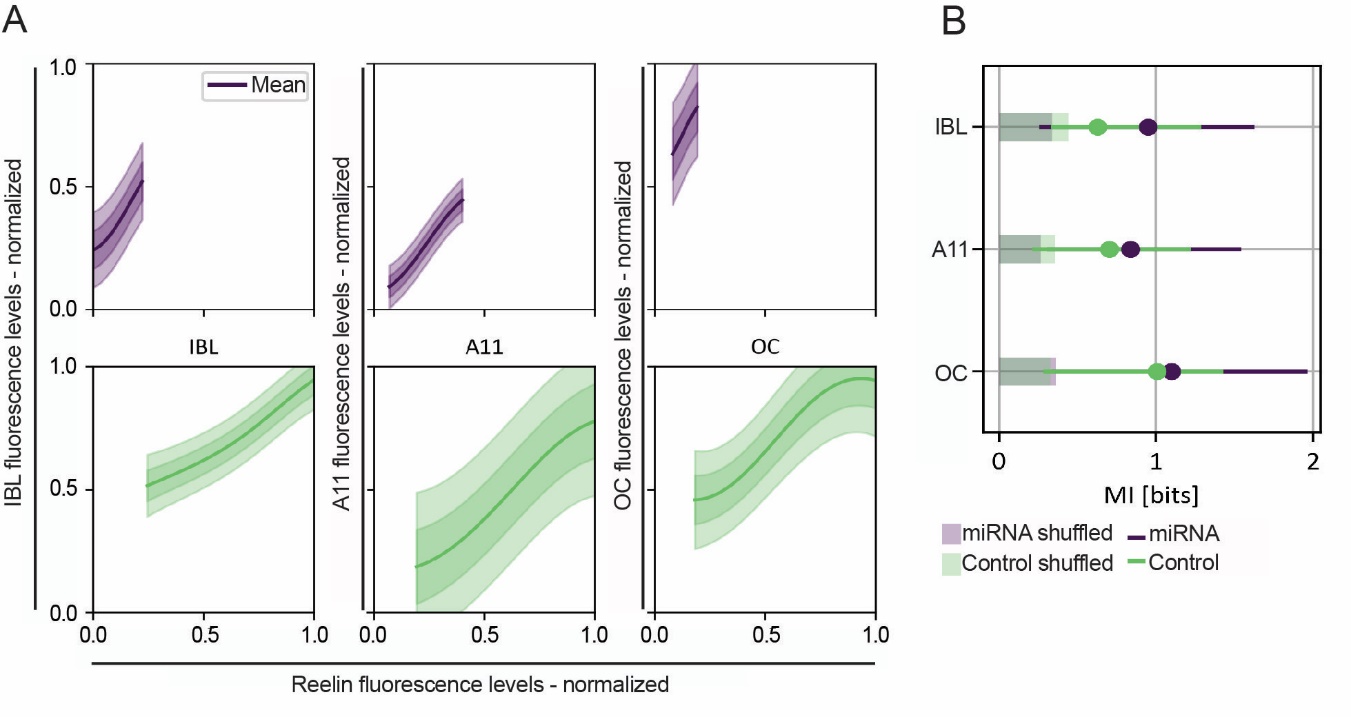


**Supplementary figure 5.** *Mutual information between* Aβ monomers/dimers (IBL), Aβ prefibrils (A11), Aβ protofibrils (OC) and reelin in Re+ alEC LII-neurons. (**A)** Posterior predictive distribution p(y|x) of iAβ levels (y-axis) vs reelin level (x-axis) (see Methods section, *Regression analysis of reelin vs iAβ fluorescence levels*) for the neurons infected by the experimental vector (miRNA-Re; top, magenta) and for the neurons infected by the control vector (bottom, cyan)*.* Each column corresponds to fluorescence data collected in all the experiments from a representative animal in which a specific form of Aβ was immunolabeled (IBL, A11, OC from left to right). Solid lines mark the mean of the distribution, darker regions the 68%ile of the credible interval and lighter regions the 95%ile of the credible interval. **(B)** Posterior distribution of mutual information MI between levels of each form of Aβ, and levels of reelin (see Methods section, *Regression analysis of reelin vs iAβ fluorescence levels*) for the neurons infected by the experimental vector (miRNA-Re, purple) and for the neurons infected by the control vector (green), pooling MI samples across animals*.* Each row corresponds to fluorescence data collected in all the experiments in which a specific form of iAβ was immunolabeled (IBL, A11, OC from top to bottom). Solid lines span the 95% credible interval, while the dots represent the mean. 95% credible interval for the shuffled data are displayed as thick semi-transparent lines. For n, see Fig. 4. Data are from five double-injected animals (see methods or Supplementary table 1).


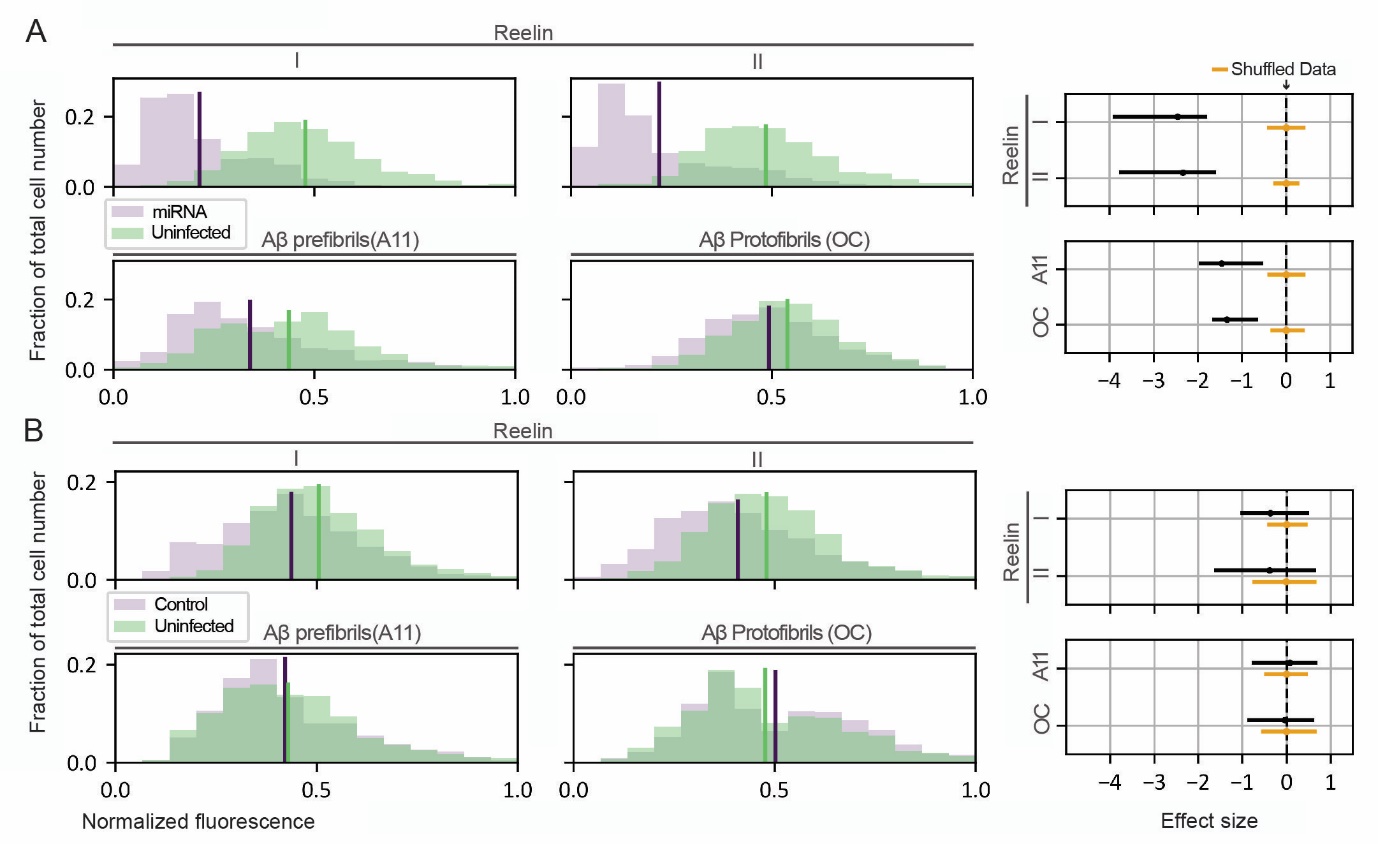


**Supplementary figure 6.** Quantification of the lowering of reelin and the concomitant reduction of iAβ in Re+ alEC LII-neurons for the second set of experiments. **(A)** Upper row: plots showing lowered levels of reelin as measured by average immunofluorescence per neuron (binned) following injections of the experimental vector (miRNA-Re: *n* I = 1601, *n* II = 1980) to lower reelin-levels, relative to uninfected Re+ neurons (*n* I = 2059, *n* II = 1892) in the contralateral alEC LII; right hand panel shows the efficacy of the manipulation expressed as posterior Cohen’s d effect sizes (mean effect size (dots) = 2.2 standard deviations, 95% credible interval (horizontal lines) = [1.7, 3.9], see Methods). Lower row: lowering reelin levels leads to a concomitant lowering of iAβ prefibrils and protofibrils. Right hand panel shows the efficacy of the manipulation (A11: mean effect size (dots) = 1.5 standard deviations, 95% credible interval (horizontal lines) = [0.5, 2.0]; OC: mean effect size = 1.3 standard deviations, 95% credible interval=[0.6, 1.7]). Posterior distribution of the mean of the effect size from the shuffled data are colored in yellow, again, solid lines span the 95% credible interval, while the dots represent the mean. **(B)** Same as for (A) but following injections of a control viral vector (control vector: *n* I = 2244, *n* II = 1178), relative to Uninfected Re+ neurons in the contralateral alEC LII (uninfected neurons: *n* I = 2219, *n* II = 1180). The control vector causes a reduction of reelin-levels (mean effect size 0.38 standard deviations, 95% credible interval [-0.6, 1.3]) relative to the uninfected neurons, but this has no effect on iAβ. Data are from 19 single side-injected animals, 9 with experimental vector, 10 with control vector (see methods or Supplementary table 1).


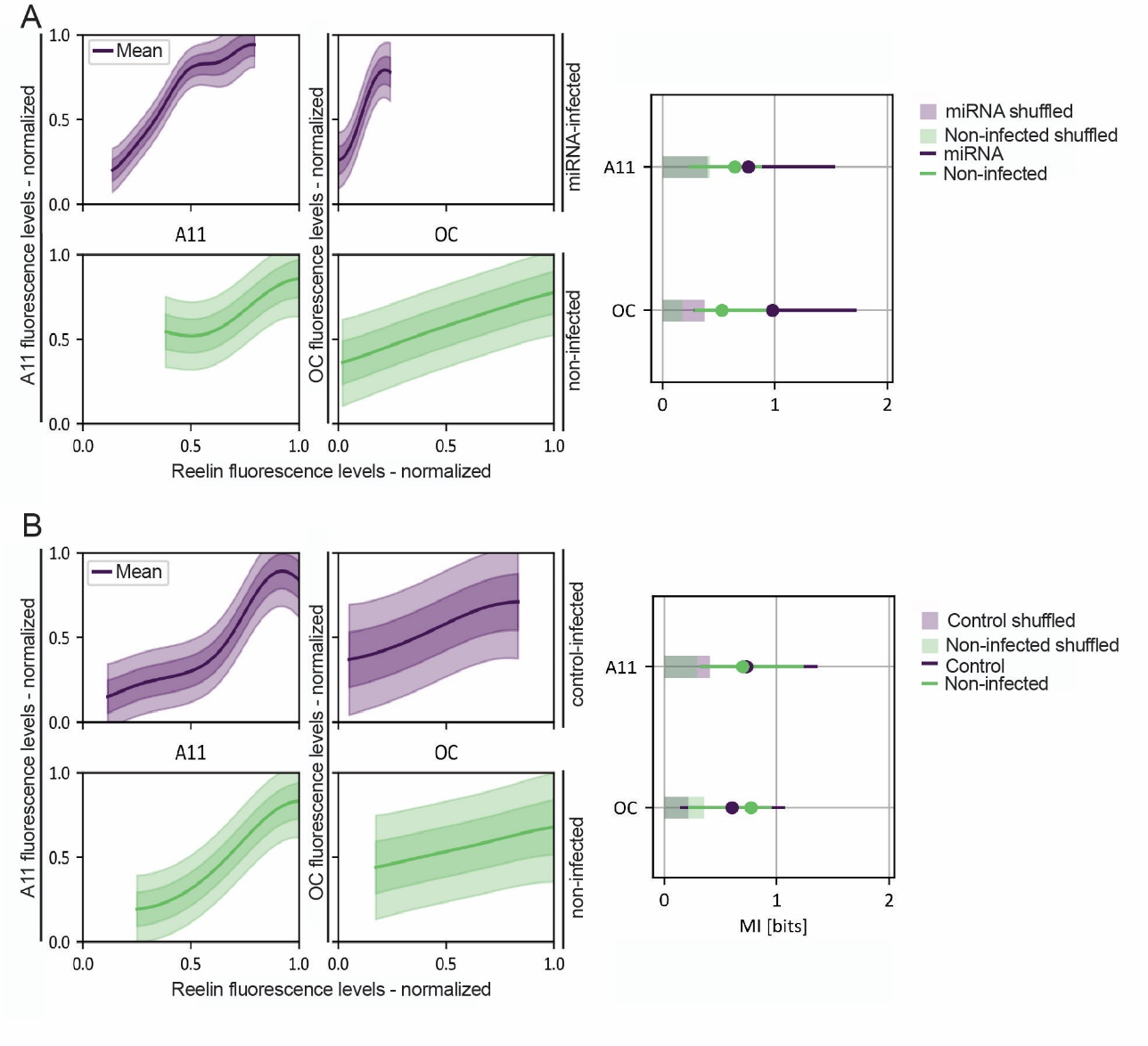


**Supplementary figure 7.** *Mutual information between* Aβ prefibrils (A11), Aβ protofibrils (OC) and reelin in Re+ alEC LII-neurons. (**A)** Left-side plots: posterior predictive distribution p(y|x) of iAβ levels (y-axis) vs reelin level (x-axis) (see Methods section, *Regression analysis of reelin vs iAβ fluorescence levels*) for the neurons infected by the experimental vector (miRNA-Re; top, purple) and for the non-infected neurons (bottom, green)*.* Each plot corresponds to fluorescence data collected in all the experiments on a representative animal in which a specific form of Aβ was immunolabelled (A11, OC from left to right). Solid lines mark the mean of the distribution, darker regions the 68%ile of the credible interval and lighter regions the 95%ile of the credible interval. Right-side schematic: Posterior distribution of mutual information MI between levels of each of the two forms of Aβ, and levels of reelin (see Methods section, *Regression analysis of reelin vs iAβ fluorescence levels*) for the neurons infected by the experimental vector (miRNA-Re, purple) and for non-infected neurons (green), pooling MI samples across animals*.* Each row corresponds to fluorescence data collected in both experiments in which the specific form of iAβ was immunolabelled (A11, OC from top to bottom). Solid lines span the 95% credible interval, while the dots represent the mean. 95% credible interval for the shuffled data are displayed as thick semi-transparent lines. **(B)** Left-side plots: posterior predictive distribution p(y|x) of iAβ levels (y-axis) vs reelin level (x-axis) for the neurons infected by the control viral vector (top, purple) and for the non-infected neurons (bottom, green)*.* Each column corresponds to fluorescence data collected in all the experiments on a representative animal in which a specific form of Aβ was immunolabelled (A11, OC from left to right). Solid lines mark the mean of the distribution, darker regions the 68%ile of the credible interval and lighter regions the 95%ile of the credible interval. Right-side schematic: Posterior distribution of mutual information MI between levels of each of the two forms of Aβ, and levels of reelin for the neurons infected by the control viral vector (purple) and for non-infected neurons (green), pooling MI samples across animals*.* Each row corresponds to fluorescence data collected in both experiments in which the specific form of iAβ was immunolabelled (A11, OC from top to bottom). Solid lines span the 95% credible interval, while the dots represent the mean. 95% credible interval for the shuffled data are displayed as thick semi-transparent lines. For *n*, see Supplementary Fig. 6.


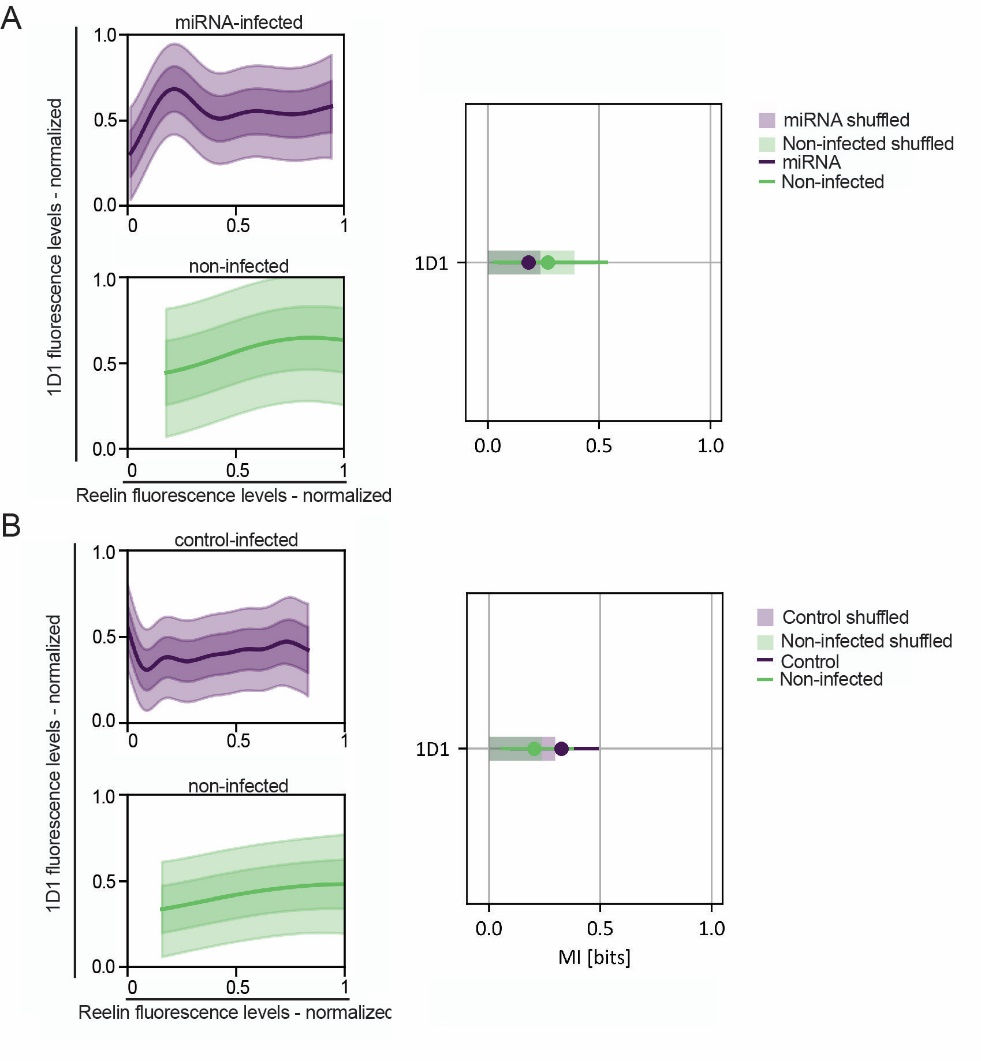


**Supplementary figure 8.** *Mutual information between* levels of human APP (hAPP; 1D1) and reelin, measured from fluorescence-levels in Re+ alEC LII-neurons. (**A)** Left-side plots: posterior predictive distribution p(y|x) of hAPP (1D1) levels (y-axis) vs reelin level (x-axis) (see Methods section, *Regression analysis of reelin vs iAβ fluorescence levels*) for the neurons infected by the experimental vector (miRNA-Re; top, purple) and for the non-infected neurons (bottom, green)*.* Both plots correspond to fluorescence data collected for the experiments from a representative animal in which hAPP (1D1) was co-immunolabelled with reelin. Solid lines mark the mean of the distribution, darker regions the 68%ile of the credible interval and lighter regions the 95%ile of the credible interval. Right-side schematic: Posterior distribution of mutual information MI between levels of hAPP and levels of reelin (see Methods section, *Regression analysis of reelin vs iAβ fluorescence levels*) for the neurons infected by the experimental vector (miRNA-Re, purple) and for non-infected neurons (green), pooling MI samples across animals*.* Solid lines span the 95% credible interval, while the dots represent the mean. 95% credible interval for the shuffled data are displayed as thick semi-transparent lines. **(B)** Left-side plots: posterior predictive distribution p(y|x) of hAPP-levels (1D1; y-axis) vs reelin level (x-axis) for the neurons infected by the control vector (top, purple) and for the non-infected neurons (bottom, green) of a representative animal. Solid lines mark the mean of the distribution, darker regions the 68%ile of the credible interval and lighter regions the 95%ile of the credible interval. Right-side schematic: Posterior distribution of mutual information MI between levels of hAPP and levels of reelin for the neurons infected by the control vector (purple) and for non-infected neurons (green), pooling MI samples across animals*.* Solid lines span the 95% credible interval, while the dots represent the mean. 95% credible interval for the shuffled data are displayed as thick semi-transparent lines. For *n*, see Fig. 5 D (double fluorescence). Data are from 19 single side-injected animals, 9 with experimental vector, 10 with control vector (see methods or Supplementary table 1).

**Supplementary table 1.** Animals used with type of injection indicated. Age/time shown in days.

|  | Animal id | Sex | age at injection | time with virus | age at termination |
| --- | --- | --- | --- | --- | --- |
| miRNA- | 24868 | male | 36 | 78 | 114 |
| infected | 24878 | Female | 39 | 75 | 114 |
|  | 26107 | Male | 37 | 60 | 97 |
|  | 26111 | Female | 42 | 55 | 97 |
|  | 26112 | Female | 42 | 55 | 97 |
|  | 26118 | Female | 43 | 54 | 97 |
|  | 26174 | Male | 35 | 59 | 94 |
|  | 26175 | Male | 35 | 59 | 94 |
|  | 26255 | Female | 24 | 58 | 82 |
| EGFP- | 26071 | Male | 24 | 61 | 85 |
| infected | 26073 | Female | 25 | 60 | 85 |
|  | 26108 | Male | 38 | 59 | 97 |
|  | 26110 | Female | 42 | 55 | 97 |
|  | 26252 | Male | 23 | 59 | 82 |
|  | 26253 | Male | 23 | 59 | 82 |
|  | 26254 | Male | 23 | 59 | 82 |
|  | 24875 | Female | 37 | 77 | 114 |
|  | 25178 | Female | 36 | 56 | 92 |
|  | 25179 | Female | 35 | 57 | 92 |
| Double- | 25180 | Female | 33 | 59 | 92 |
| infected | 25181 | Male | 33 | 59 | 92 |
|  | 25561 | Female | 32 | 63 | 95 |
|  | 25562 | Female | 33 | 62 | 95 |
|  | 24879 | Female | 38 | 76 | 114 |

**Supplementary table 2.** Overview of primary and secondary antibodies used. Note that we co-labeled with anti-reelin (leftmost column) for each of the other primary antibodies. Permeabilization agents used: *0.4% Saponin (VWR, Cat# 27534.187); **0.2% Triton-X100 (Millipore, Cat# 1.08603.1000). The anti-hAPP 1D1 antibody was a gift from Steffen Rossner at Paul Flechsig Institute for Brain Research, University of Leipzig, Germany20; for this antibody we co-blocked (together with goat serum) with 3% donkey-Fab anti-rat IgG (Jackson ImmunoResearch Labs Cat# 712-007-003 RRIDAB_2340634).

| Primary antibodies  ______________________________________________ | | | | | | |  | Secondary antibodies  _______________________________________ | | | |
| --- | --- | --- | --- | --- | --- | --- | --- | --- | --- | --- | --- |
|  |  | Type/ dilution | Host | Name/ clone_ | Vendor | Time/  temp |  | Goat a-rabbit 546, Cat# A-11010, RRID: AB_2534077 | Goat a-mouse 635, Cat# A-11010, RRID: AB_2534077 | Goat a-rat 546, Cat# A- 11081, RRID: AB_ 2534125 | Time/  temp |
|  |  |  |  |  |  |  |  |  |  |  |  |
|  |  |  |  |  |  |  |  |  |  |  |  |
| anti-Re  (1:1000)  Host = Mouse Clone G10  Vendor: Millipore_  Cat#MAB5364RRID:AB_2179313 |  | anti-Aβ42/ 1:1000** | rabbit | IBL  Aβ42 | IBL Ltd. Cat#18582 RRID:AB_2341375 | 4_hrs/ 4_C° |  | 🗸 | 🗸 |  | 2_hrs/ ~20C° |
|  |  | anti-Oligome/ 1:1000* | rabbit | A11 | Thermo_Fisher Cat#AHB0052_ RRID:AB_ 2536236 | 20_hrs/ 4_C° |  | 🗸 | 🗸 |  | 2_hrs/ ~20C° |
|  |  | anti-Protofib./ 1:1000* | rabbit | OC | Millipore_Cat# AB2286, RRID: AB_1977024 | 20_hrs/ 4_C° |  | 🗸 | 🗸 |  | 2_hrs/ ~20C° |
|  |  | anti-hAPP/ 1:3** | rat | 1D1 | Gift6from S._Rösner | 40_hrs/ 4_C° |  |  | 🗸 | 🗸 | 2_hrs/ ~20C° |
|  |  | anti- calbindin/ 1:1000** | Mouse | - | Swant  Cat#300  RRID: AB_10000347 | 40_hrs/ 4_C° |  | 🗸 | 🗸 |  | 2_hrs/ ~20C° |
